# Quantifying the value of surveillance data for improving model predictions of lymphatic filariasis elimination

**DOI:** 10.1371/journal.pntd.0006674

**Published:** 2018-10-08

**Authors:** Edwin Michael, Swarnali Sharma, Morgan E. Smith, Panayiota Touloupou, Federica Giardina, Joaquin M. Prada, Wilma A. Stolk, Deirdre Hollingsworth, Sake J. de Vlas

**Affiliations:** 1 Department of Biological Sciences, University of Notre Dame, Notre Dame, South Bend, IN, United States of America; 2 Department of Statistics, University of Warwick, Coventry, United Kingdom; 3 Department of Public Health, Erasmus MC, University Medical Center Rotterdam, Rotterdam, Netherlands; 4 Faculty of Health & Medical Sciences, University of Surrey, Guildford, United Kingdom; 5 Big Data Institute, University of Oxford, Oxford, United Kingdom; Royal Veterinary College, UNITED KINGDOM

## Abstract

**Background:**

Mathematical models are increasingly being used to evaluate strategies aiming to achieve the control or elimination of parasitic diseases. Recently, owing to growing realization that process-oriented models are useful for ecological forecasts only if the biological processes are well defined, attention has focused on data assimilation as a means to improve the predictive performance of these models.

**Methodology and principal findings:**

We report on the development of an analytical framework to quantify the relative values of various longitudinal infection surveillance data collected in field sites undergoing mass drug administrations (MDAs) for calibrating three lymphatic filariasis (LF) models (EPIFIL, LYMFASIM, and TRANSFIL), and for improving their predictions of the required durations of drug interventions to achieve parasite elimination in endemic populations. The relative information contribution of site-specific data collected at the time points proposed by the WHO monitoring framework was evaluated using model-data updating procedures, and via calculations of the Shannon information index and weighted variances from the probability distributions of the estimated timelines to parasite extinction made by each model. Results show that data-informed models provided more precise forecasts of elimination timelines in each site compared to model-only simulations. Data streams that included year 5 post-MDA microfilariae (mf) survey data, however, reduced each model’s uncertainty most compared to data streams containing only baseline and/or post-MDA 3 or longer-term mf survey data irrespective of MDA coverage, suggesting that data up to this monitoring point may be optimal for informing the present LF models. We show that the improvements observed in the predictive performance of the best data-informed models may be a function of temporal changes in inter-parameter interactions. Such best data-informed models may also produce more accurate predictions of the durations of drug interventions required to achieve parasite elimination.

**Significance:**

Knowledge of relative information contributions of model only versus data-informed models is valuable for improving the usefulness of LF model predictions in management decision making, learning system dynamics, and for supporting the design of parasite monitoring programmes. The present results further pinpoint the crucial need for longitudinal infection surveillance data for enhancing the precision and accuracy of model predictions of the intervention durations required to achieve parasite elimination in an endemic location.

## Author summary

Although parasite transmission models offer powerful tools for predicting the impacts of interventions, there is growing realization that these models can be useful for this purpose only if their governing biological processes are well defined. Recently, model-data assimilation has been applied to address this problem and improve the performance of process-oriented models for ecological forecasting. Here, we developed an analytical framework that allowed the sequential coupling of the three existing lymphatic filariasis (LF) models with longitudinal infection monitoring data collected in field sites undergoing mass drug administrations (MDAs) to examine the relative value of such data for parameterizing these models and for improving their predictions of the required durations of drug interventions to break parasite transmission. We found that data-informed models provided more precise and reliable forecasts of elimination timelines in the study sites compared to model-only predictions, and that data collected up to 5 years post-MDA reduced each model’s predictive uncertainty most. We also found that this improved performance may be intriguingly related to temporal changes in system dynamics. Our results underscore the significance of sequential model-data fusion for enhancing the understanding of LF transmission dynamics, design of surveillance, and generation of reliable model predictions for management decision making.

## Introduction

Mathematical models of parasite transmission, via their capacity for producing dynamical forecasts or predictions of the likely future states of an infection system, offer an important tool for guiding the development and evaluation of strategies aiming to control or eliminate infectious diseases [1–7]. The power of these numerical simulation tools is based uniquely on their ability to appropriately incorporate the underlying nonlinear and multivariate processes of pathogen transmission in order to facilitate plausible predictions outside the range of conditions at which these processes are either directly observed or quantified [8–11]. The value of these tools for guiding policy and management decisions by providing comparative predictions of the outcomes of various strategies for achieving the control or elimination of the major Neglected Tropical Diseases (NTDs) has been highlighted in a series of recent publications [8, 11, 12], demonstrating the crucial role these quantitative tools are beginning to play in advancing policy options for these diseases.

While these developments underscore the utility of transmission models for supporting policy development in parasite control, a growing realization is that these models can be useful for this purpose only if the biological processes are well defined and demographic and environmental stochasticity are either well-characterized or unimportant for meeting the goal of the policy modelling exercise [9–11, 13–16]. This is because the realized predictability of any model for a system depends on the initial conditions, parameterizations and process equations that are utilized in its simulation such that model outcomes are strongly sensitive to the choice of values used for these variables [17]. Any misspecification of these system attributes will lead to failure in accurately forecasting the future behaviour of a system, with predictions of actual future states becoming highly uncertain even when the exact representation of the underlying deterministic process is well established but precise specification of initial conditions or forcing and/or parameter values is difficult to achieve [17, 18]. This problem becomes even more intractable when theoretical models depend on parameter estimates taken from other studies [5, 17, 19]. Both these challenges, viz. sensitivity to forcing conditions and use of parameter estimates from settings that are different from the dynamical environment in which a model will be used for simulation, imply that strong limits will be imposed on the realized predictability of any given model for an application [9, 10, 20]. As we have shown recently, if such uncertainties are ignored, the ability of parasite transmission models to form the scientific basis for management decisions can be severely undermined, especially when predictions are required over long time frames and across heterogeneous geographic locations [4, 5, 7].

These inherent difficulties with using an idealized model for producing predictions to guide management have led to consideration of data-driven modelling procedures that allow the use of information contained within observations to improve specification and hence the predictive performance of process-based models [9, 10, 14, 21–23]. Such approaches, termed model-data fusion or data assimilation methods, act by combining models with various data streams (including observations made at different spatial or temporal scales) in a statistically rigorous way to inform initial conditions, constrain model parameters and system states, and quantify model errors. The result is the discovery of models that can more adequately capture the prevailing system dynamics in a site, an outcome which in turn has been shown to result in the making of significantly improved predictions for management decision making [9, 10, 14, 24]. Initially used in geophysics and weather forecasting, these methods are also beginning to be applied in ecological modelling, including more recently in the case of infectious disease modelling [9, 10]. In the latter case, the approach has shown that it can reliably constrain a disease transmission model during simulation to yield results that approximate epidemiological reality as closely as possible, and as a consequence improve the accuracy of forecasts of the response of a pathogen system exposed to various control efforts [4–7, 21, 25–27].

More recently, attention has also focused on the notion that a model essentially represents a conditional proposition, i.e. that running a model in a predictive mode presupposes that the driving forces of the system will remain within the bounds of the model conceptualization or specification [28]. If these driving forces were to change, then it follows that even a model well-calibrated to a given historical dataset will fail. New developments in longitudinal data assimilation can mitigate this problem of potential time variation of parameters via the recursive adjustment of the model by assimilation of data obtained through time [22, 29, 30]. Apart from allowing assessment of whether stasis bias may occur in model predictions, such sequential model calibration with time-varying data can also be useful for quantifying the utility of the next measurement in maximizing the information gained from all measurements together [31]. Carrying out such longitudinal model-data analysis has thus the potential for providing information to improve the efficiency and cost-effectiveness of data monitoring campaigns [24, 31–33], along with facilitating more reliable model forecasts.

A key question, however, is evaluating which longitudinal data streams provide the most information to improve model performance [33]. Indeed, it is possible that from a modelling perspective using more data may not always lead to a better-constrained model [34]. This suggests that addressing this question is not only relevant to model developers, who need observational data to improve, constrain, and test models, but also for disease managers working on the design of disease surveillance plans. At a more philosophical level, we contend that these questions have implications for how current longitudinal monitoring data from parasite control programmes can best be exploited both scientifically and in management [31]. Specifically, we suggest that these surveillance data need to be analysed using models in a manner that allows the extraction of maximal information about the monitored dynamical systems so that this can be used to better guide both the collection of such data as well as the provision of more precise estimates of the system state for use in making state-dependent decisions [2, 35–37]. Currently, parasite control programmes use infection monitoring data largely from sentinel sites primarily to determine if an often arbitrarily set target is met [3]. Little consideration is given to whether these data could also be used to learn about the underlying transmission dynamics of the parasitic system, or how such learning can be effectively used by management to make better decisions regarding the interventions required in a setting to meet stated goals [2, 4].

Here, we develop an analytical framework to investigate the value of using longitudinal LF infection data for improving predictions of the durations of drug interventions required for achieving LF elimination by coupling data collected during mass drug interventions (MDAs) carried out in three example field sites to three existing state-of-the-art lymphatic filariasis (LF) models [4, 6, 21, 38–43]. To be managerially relevant to current WHO-specified LF intervention surveillance efforts, we evaluated the usefulness of infection data collected in these sites at the time points proposed by the WHO monitoring framework in carrying out the present assessment [44]. This was specifically performed by ranking these different infection surveillance data streams according to the incremental information gain that each stream provided for reducing the prediction uncertainty of each model.

## Methods

### Data

Longitudinal pre- and post-infection and MDA data from representative sites located in each of the three major regions endemic for LF (Africa, India, and Papua New Guinea (PNG)) were assembled from the published literature for use in constraining the LF models employed in this study. The three sites (Kirare, Tanzania, Alagramam, India, and Peneng, PNG) were selected on the basis that each represents the average endemic transmission conditions (average level of infection, transmitting mosquito genus) of each of these three major extant LF regions, while providing details on the required model inputs and data for conducting this study. These data inputs encompassed information on the annual biting rate (ABR) and dominant mosquito genus, as well as MDA intervention details, including the relevant drug regimen, time and population coverage of MDA, and times and results of the conducted microfilaria (mf) prevalence surveys (Table 1). Note each site also provided these infection and MDA data at the time points pertinent to the existing WHO guidelines for conducting LF monitoring surveys during a MDA programme [44], which additionally, as pointed out above, allowed the assessment of the value of such infection data both for supporting effective model calibration and for producing more reliable intervention forecasts.

**Table 1:** Annual mf prevalence survey and MDA data for three LF endemic sites.

*Village*	*Kirare, Tanzania [45]*			*Alagramam, India [46]*			*Peneng, PNG [6]*		
*Regimen* *(efficacy^a^)*	IVM+ALB (99/9)			DEC (90/3)			DEC+IVM (99/9)		
*Mosquito Genus*	Anopheles^b^			Culex			Anopheles		
*ABR^c^*	2090^b^			20000 [47]			8194		
	Year (Survey/ MDA)^d^	Mf Prev (No. sampled)	Total Population MDA Cov.^f^	Year^d^	Mf Prev (No. sampled^g^)	Total Population MDA Cov.	Year^d^	Mf Prev (No. sampled)	Total Population MDA Cov.
***Pre-treatment***	Sept 2004/ Oct 2004	26.1% (471)	72%	Nov 1994	17.2% (230)	48%^h^	1994	66.7% (63)	50%
***Mid-treatment (Post-MDA 1–4)***	Jan 2006/ Feb 2006	20.8% (461)	70%	May 1995	18.5% (230)	48%^h^	1995	61.5% (65)	78%
	Jan 2007/ May 2007	15.8% (438)	62%	Aug 1996	14.5% (230)	48%^h^	1996	20.5% (88)	75%
	Oct 2008/ Feb 2009	12.9% (302)	59%	Nov 1997	11.8% (230)	48%^h^	1997	13.5% (89)	68%
	Oct 2009/ Nov 2009	5.0% (259)	76%	Feb 1999	12.2% (230)	48%^h^	1998	5.4% (92)	72%
***Late-treatment (Post-MDA 5+)***	Nov 2010/ Dec 2010	4.4% (400)^e^	60%	April 2000	4.9% (230)	48%^h^	1999	3.7% (109)	-
	Nov 2011	2.7% (393)^e^	**-**	April 2001	4.2% (230)	-	-	-	-

^a^Drug efficacy assumptions are listed as instantaneous mf kill rate/duration of sterilization in months [1]

^b^Transmission in Kirare is by both Anopheles and Culex mosquitoes, but models based on the dominant species (Anopheles) were used in this study. The ABR represents the combined biting rate [45].

^c^In the model simulations, the allowed ABR range was informed by the observed ABRs reported here.

^d^The “Mf Prev” columns denote the prevalence for a given year which was surveyed right before the MDA given in that year at the coverage reported in the column “MDA Cov”. Some mid-treatment surveys in Kirare, Tanazania do not follow this pattern exactly, so for that site the time of the mf survey and the time of the treatment of that year are given explicitly. The survey and MDA times are reflected in the model simulations.

^e^The number tested represented those tested for CFA. Only those positive for CFA were tested for mf. The expected number of mf positives in the total sample were calculated as [number positive for CFA]x[number positive for mf]/[number of CFA positives examined for mf] as given in [45].

^f^The total coverage was calculated using annual population sizes and coverage of the eligible population (≥ 5 years old) given in [45] and the fraction of individuals ≥ 5 years old calculated from [48].

^g^The number of individuals sampled is reported as a random 7% of households which we assume here to represent 7% of the total population.

^h^MDA coverage reported as ranging between 50–71% of the eligible population throughout the programme in [46]. The average total population coverage calculated as [average coverage]x[proportion of the population eligible for treatment] based on figures given in [46] was modelled.

### The models

The three existing LF models employed for this study included EPIFIL, a deterministic Monte Carlo population-based model, and LYMFASIM and TRANSFIL, which are both stochastic, individual-based models. All three models simulate LF transmission in a population by accounting for key biological and intervention processes such as impacts of vector density, the life cycle of the parasite, age-dependent exposure, density-dependent transmission processes, infection aggregation, and the effects of drug treatments as well as vector control [4, 21, 38–40, 42, 43, 49]. Although the three models structurally follow a basic coupled immigration-death model formulation, they differ in implementation (e.g. from individual to population-based), the total number of parameters included, and the way biological and intervention processes are mathematically incorporated and parameterized. The three models have been compared in recent work [8, 12], with full details of the implementation and simulation procedures for each individual model also described [6, 8, 12, 21, 39, 42, 43, 49, 50]. Individual model parameters and fitting procedures specific to this work are given in detail in S1 Supplementary Information.

### Longitudinal data assimilation procedures

We used longitudinal data assimilation methods to sequentially calibrate the three LF models with the investigated surveillance data such that parameter estimates and model predictions reflect not only the information contained in the baseline but also follow-up data points. The available mf prevalence data from each site were arranged into four different temporal data streams to imitate the current WHO guidelines regarding the time points for conducting monitoring surveys during an MDA programme. This protocol proposes that infection data be collected in sentinel sites before the first round of MDA to establish baseline conditions, no sooner than 6 months following the third round of MDA, and no sooner than 6 months following the fifth MDA to assess whether transmission has been interrupted (defined as reduction of mf prevalence to below 1% in a population) [44, 51]. Thus, the four data streams considered for investigating the value of information gained from each survey were respectively: scenario 1—baseline mf prevalence data only, scenario 2—baseline and post-MDA 3 mf prevalence data, scenario 3—baseline, post-MDA 3, and post-MDA 5 mf prevalence data, and scenario 4—baseline and post-MDA 5 mf prevalence data. In addition to these four data streams, a fifth model-only scenario (scenario 0) was also considered where no site-specific data was introduced. In this case, simulations of interventions were performed using only model-specific parameter and ABR priors estimated for each region.

The first step for all models during the data assimilation exercises reported here was to initially simulate the baseline infection conditions in each site using a large number of samples (100,000 for EPIFIL and TRANSFIL, and 10,000–30,000 for LYMFASIM) randomly selected from the parameter priors deployed by each model. The number of parameters which were left free to be fitted to these data by each model range from 3 (LYMFASIM and TRANSFIL) to 21 (EPIFIL). The ABR, a key transmission parameter in all three models, was also left as a free parameter whose distribution was influenced by the observed ABR (Table 1) and/or by fits to previous region-specific datasets (see S1 Supplementary Information for model-specific implementations). The subsequent steps used to incorporate longitudinal infection data into the model calibration procedure varied among the models, but in all cases the goodness-of-fit of the model outputs for the site-specific mf prevalence data was assessed using the chi-square metric (α = 0.05) [52].

**Fig 1:**
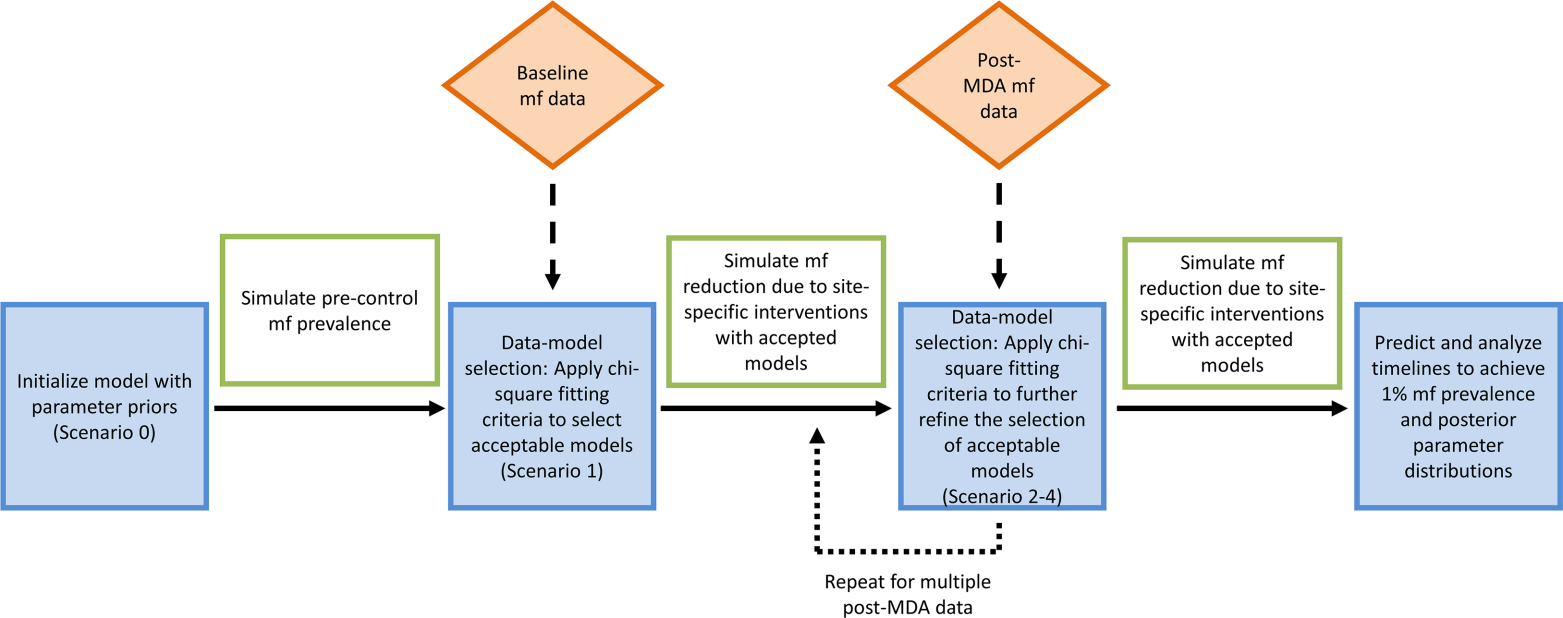
Schematic diagram showing the sequential fitting procedure for updating models and predictions by incorporating longitudinal data. In all scenarios, the initial EPIFIL models were initialized with parameter priors and a chi-square fitting criterion was applied to select those models which represent the baseline mf prevalence data sufficiently well (α = 0.05). The accepted models were then used to simulate the impact of interventions on mf prevalence. The chi-square fitting criterion was sequentially applied to refine the selection of models according to the post-MDA mf prevalence data included in the fitting scenario. The fitted parameters from selection of acceptable models at each data point were used to predict timelines to achieve 1% mf prevalence. The scenarios noted in the blue boxes indicate the final relevant updating step before using the fitted parameters to predict timelines to achieve 1% mf in that data fitting scenario.

EPIFIL used a sequential model updating procedure to iteratively modify the parameters with the introduction of each subsequent follow up data point through time [6]. This process uses parameter estimates from model fits to previous data as priors for the simulation of the next data which are successively updated with the introduction of each new observation, thus providing a flexible framework by which to constrain a model using newly available data. Fig 1 summarizes the iterative algorithm used for conducting this sequential model-data assimilation exercise [6]. LYMFASIM and TRANSFIL, by contrast, included all the data in each investigated stream together for selecting the best-fitting models for each time series–i.e. model selection for each data series was based on using all relevant observations simultaneously in the fitting process [30, 53, 54]. Although a limitation of this batch estimation approach is that the posterior probability of each model is fixed for the whole simulation period, unlike the case in sequential data assimilation where a restricted set of parameters is exposed to each observation (as a result of parameter constraining by data used in the previous time step)–which thereby yields models that give better predictions for different portions of the underlying temporal process—here we use both methods to include and assess the impact that this implementation difference may have on the results presented below. For all models, the final updated parameter estimates from each data stream were used to simulate the impact of observed MDA rounds and for predicting the impact of continued MDA to estimate how many years were required to achieve 1% mf prevalence.

### Intervention modelling

Interventions were modelled by using the updated parameter vectors or models selected from each scenario for simulating the impact of the reported as well as hypothetical future MDA rounds on the number of years required to reduce the observed baseline LF prevalence in each site to below the WHO transmission threshold of 1% mf prevalence [44]. When simulating these interventions, the observed MDA times, regimens, and coverages followed in each site were used (Table 1), while MDA was assumed to target all residents aged 5 years and above. For making mf prevalence forecasts beyond the observations made in each site, MDA simulations were extended for a total of 50 annual rounds in each site at an assumed coverage of 65%. While the drug-induced mf kill rate and the duration of adult worm sterilization were fixed among the models (Table 1), the worm kill rate was left as a free parameter to be estimated from post-intervention data to account for the uncertainty in this drug efficacy parameter [4, 7, 21]. The number of years of MDA required to achieve the threshold of 1% mf prevalence was calculated from model forecasts of changes in mf prevalence due to MDA for each model-data fusion scenario.

### Information contribution of model and data

The predictions from each model regarding timelines to achieve 1% mf for each fitting scenario were used to determine the information gained from each data stream compared to the information attributable to the model itself [14, 33, 55]. The relative information gained from a particular data stream was calculated as *I*_*d*_ *= H*_*m*_*—H*_*md*_ where *H* measures the entropy or uncertainty associated with a random variable, *H*_*m*_ denotes predictions from the model-only scenario (scenario 0) which essentially represents the impact of prior knowledge of the system, and *H*_*md*_ signifies predictions from each of the four model-data scenarios (i.e. scenarios 1–4). The values of *I*_*d*_ for each data scenario or stream were compared in a site to infer which survey data are most useful for reducing model uncertainty. The Shannon information index was used to measure entropy, *H*, as follows: $H=-\sum_{i=1}^{m}p(x_{i}){log}_{2}p(x_{i})$, where *p(x*_*i*_*)* is the discrete probability density function (PDF) of the number of years of MDA predicted by each fitted model to reach 1% mf, and is estimated from a histogram of the respective model predictions for *m* bins (of equal width in the range between the minimum and maximum values of the PDFs) [14, 56].

To statistically compare two entropy values, a permutation test using the differential Shannon entropy (*DSE*) was performed [57]. *DSE* is defined as |*H*_*1*_*—H*_*2*_| where *H*_*1*_ was calculated from the distribution of timelines to achieve 1% mf for a given scenario, *y*_*1*_, and *H*_*2*_ was calculated from the distribution of timelines to achieve 1% mf for a different scenario, *y*_*2*_. The list of elements in *y*_*1*_ and *y*_*2*_ were combined into a single list of size *y*_*1*_ + *y*_*2*_ and the list was permuted 20,000 times. *DSE* was then recalculated each time by calculating a new *H*_*1*_ from the first *y*_*1*_ elements and a new *H*_*2*_ from the last *y*_*2*_ elements from each permutation, from which *p*-values may be quantified as the proportion of all recalculated *DSE*s that were greater than the original *DSE*.

### Weighting model predictions

Model predictions of the mean and variance in timelines to LF elimination were weighted according to the frequencies by which predictions occurred in a group of simulations. In general, if *D*_1_,*D*_2_,…,*D_n_* are data points (model predictions in the present case) that occur in an ensemble of simulations with different weights or frequencies *W*_1_,*W*_2_,…,*W_n_*, then the weighted mean, *Wmean*, = $\frac{\sum_{i=1}^{n}W_{i}*D_{i}}{\sum_{i=1}^{n}W_{i}}$, while the weighted variance, *Wvariance*, = $\frac{\sum_{i=1}^{n}W_{i}*{\left(D_{i}-Wmean\right)}^{2}}{\frac{(n^{\prime }-1)*\sum_{i=1}^{n}W_{i}}{n^{\prime }}}$ Here, *n* is the number of data points and *n*′ is the number of non-zero weights. In this study, the weighted variance of the distributions of predicted timelines to achieve 1% mf prevalence was calculated to provide a measure of the precision of model predictions in addition to the entropy measure, *H*. A similar weighting scheme was also used to pool the timeline predictions of all three models. Here, predictions made by each of the three models for each data scenario were weighted as above, and a composite weighted 95% percentile interval for the pooled predictions was calculated for each data stream. This was done by first computing the weighted percentiles for the combined model simulations from which the pooled 2.5^th^ and 97.5^th^ percentile values were quantified. The Matlab function, *wprctile*, was used to carry out this calculation.

### Parameter constraints and interactions

The extent by which parameter constraints are achieved through the coupling of models with data was evaluated to determine if improvements in such constraints by the use of additional data may lead to reduced model prediction uncertainty [33]. Parameter constraint was calculated as the ratio of the mean standard deviation of all fitted parameter distributions to the mean standard deviation of all prior parameter distributions. A ratio of less than one indicates the fitted parameter space is more constrained than the prior parameter space [33]. This assessment was carried out using the EPIFIL model only. In addition, pairwise parameter correlations were also evaluated to assess whether the sign, magnitude, and significance of these correlations changed by scenario to determine if using additional data might alter these interactions to better constrain a model. For this assessment, Spearman’s correlation coefficients and *p-*values testing the hypothesis of no correlation against the alternative of correlation were calculated, and the exercise was run using the estimated parameters from the EPIFIL model.

### Sensitivity analyses

EPIFIL was used to conduct a sensitivity analysis investigating whether the trend in relative information gained by coupling the model with longitudinal data was dependent on the interventions simulated. The same series of simulations (for three LF endemic sites and five fitting scenarios) were completed with the extended MDA coverage beyond the observations given in Table 1 set here at 80% instead of 65% to represent an optimal control strategy. As before, the timelines to reach 1% mf prevalence in each fitting scenario were calculated and used to determine which data stream provided the model with the greatest gain of information. The results were compared to the original series of simulations to assess whether the trends are robust to changes in the intervention coverages simulated.

EPIFIL was also used to perform another sensitivity analysis expanding the number of data streams to investigate if the WHO monitoring scheme is adequate for informing the making of reliable model-based predictions of timelines for achieving LF elimination. To perform this sensitivity analysis, pre- and post-MDA data from Villupuram district, India that provide extended data points (viz. scenario 1–4 as previously defined, plus scenario 5—baseline, post-MDA 3, post-MDA 5, and post-MDA 7 mf prevalence data, and scenario 6—baseline, post-MDA 3, post-MDA 5, post-MDA 7, and post-MDA 9 mf prevalence) were assembled from the published literature [47, 58]. The timelines to reach 1% mf prevalence and the entropy for each of these additional scenarios were calculated to determine whether additional data streams over those recommended by WHO are required for achieving more reliable model constraints, which among these data might be considered as compulsory, and which might be optional for supporting predictions of elimination.

### Statistical analyses

Differences in predicted medians, weighted variances and entropy values between data scenarios, models and sites were statistically evaluated using Kruskall-Wallis tests for equal medians, *F-*tests for equality of variance, and *DSE* permutation tests, respectively. *P*-values for assessing significance for all pairwise tests were obtained using the Benjamini-Hochberg procedure for controlling the false discovery rate, i.e. for protecting against the likelihood of obtaining false positive results when carrying out multiple testing [59].

## Results

### Assessing the benefit of longitudinal monitoring data for modelling LF elimination

Here, our goal was twofold. First, to determine if data are required to improve the predictability of intervention forecasts by the present LF models in comparison with the use of theoretical models only, and second, to evaluate the benefit of using different longitudinal streams of mf survey data for calibrating the three models in order to determine which data stream was most informative for reducing the uncertainty in model predictions in a site. Table 2 summarises the key results from our investigation of these questions: these are the number of accepted best-fitting models for each data stream or scenario in the three study sites (Table 1), the predicted median and range (2.5^th^-97.5^th^ percentiles) in years to achieve the mf threshold of 1% mf prevalence, the weighted variance and entropy values based on these predictions, and the relative information gained (in terms of reduced prediction uncertainty) by the use of longitudinal data for constraining the projections of each of the three LF models investigated. Even though the number of selected best-fit models based on the chi-square criterion (see Methods) differed for each site and model, these results indicate unequivocally that models constrained by data provided significantly more precise intervention predictions compared to model-only predictions (Table 2). Note that this was also irrespective of the two types of longitudinal data assimilation procedures (sequential vs. simultaneous) used by the different models in this study. Thus, for all models and sites, model-only predictions made in the absence of data (scenario 0) showed the highest prediction uncertainty, highlighting the need for data to improve the predictive performance of the present models. The relative information gained by using each data stream in comparison to model-only predictions further support this finding, with the best gains in reducing model prediction uncertainty provided by those data constraining scenarios that gave the lowest weighted variance and entropy values; as much as 92% to 96% reductions in prediction variance were achieved by these scenarios in comparison to model-only predictions between the three models (Table 2). The results also show, however, that data streams including post-MDA 5 mf survey data (scenarios 3 and 4) reduced model uncertainty (based on both the variance and entropy measures) most compared to data streams containing only baseline and/or post-MDA 3 mf survey data (scenarios 1 and 2) (Table 2). Although there were differences between the three models (due to implementation differences either in how the models are run (Monte Carlo deterministic vs. individual-based) or in relation to how the present data were assimilated (see above)), overall, scenario 3, which includes baseline, post-MDA 3, and post-MDA 5 data, was shown to most often reduce model uncertainty the greatest. Additionally, there was no statistical difference between the performances of scenarios 3 and 4 in those cases where scenario 4 resulted in the greatest gain of information (Table 2). It is also noticeable that the best constraining data stream for each combination of site and model also produced as expected the lowest range in predictions of the numbers of years of annual MDA required to achieve the 1% mf prevalence in each site, with the widest ranges estimated for model-only predictions (scenario 0) and the shorter data streams (scenario 1). In general, this constriction in predictions also led to lower estimates of the median times to achieve LF elimination, although this varied between models and sites (Table 2).

**Table 2:** Model predictions of timelines to achieve 1% mf prevalence and corresponding information metrics.

Model	Site	Scenario^#^	No. of accepted models	Median no. of years (2.5^th^-97.5^th^ percentiles) ^(significance^*^)^	Weighted variance ^(significance^*^)^	Entropy ^(significance^*^)^	Relative information gained by data (%)^+^
EPIFIL	Kirare	0	865	9 (6–19)^1,2,3,4^	14.71^1,2,3,4^	3.51^1,2,3,4^	-
		1	829	8 (6–17)^0,2,3,4^	10.37^0,3,4^	3.13^0,2,3,4^	12.06
		2	117	14 (11–21)^0,1^	8.66^0,4^	3.27^0,1,4^	6.84
		3	105	14 (11–18)^0,1^	5.82^0,1^	3.06^0,1,4^	12.82
		**4**	**175**	**12 (10–18)**^**0,1**^	**5.73**^**0,1,2**^	**2.92**^**0,1,2,3**^	**16.81**
	Alagramam	0	15098	10 (7–23)^1,2,3,4^	19.62^1,2,3,4^	3.69^1,2,3,4^	-
		1	16410	9 (7–21) ^0,2,3,4^	14.35^0,3,4^	3.53^0,2,3,4^	4.34
		2	11026	11 (8–22)^0,2,3,4^	14.44^0,3,4^	3.59^0,1,3,4^	2.71
		3	10351	11 (8–19)^0,1,2,4^	9.60^0,1,2,4^	3.38^0,1,2,4^	8.4
		**4**	**15735**	**9 (7–18)**^**0,1,2,3**^	**10.03**^**0,1,2,3**^	**3.36**^**0,1,2,3**^	**8.94**
	Peneng	0	4610	12 (6–29)^1,2,3,4^	38.24^1,2,3,4^	4.29^1,2,3,4^	-
		1	4255	10 (6–25)^0,2,3,4^	26.92^0,2,3,4^	4.02^0,2,3,4^	6.29
		2	2714	10 (7–17)^0,1,3,4^	8.37^0,1,3,4^	3.29^0,1,3,4^	23.31
		**3**	**2172**	**9 (7–12)**^**0,1,2,4**^	**3.04**^**0,1,2,4**^	**2.64**^**0,1,2,4**^	**38.46**
		4	2728	8 (6–12)^0,1,2,3^	3.86^0,1,2,3^	2.8^0,1,2,3^	34.73
LYMFASIM	Kirare	0	6471	11 (7–28)^1,2^	35.31^1,2,3,4^	4.19^2,3,4^	-
		1	901	10 (6–34)^0,2^	50.91^0,2,3,4^	4.20^2,3,4^	-0.24
		2	363	13 (10–20)^0,1,3,4^	9.50^0,3,4^	3.31^0,1,3,4^	21.00
		**3**	**224**	**11 (9–14)**^**2**^	**1.87**^**0,1,2**^	**2.39**^**0,1,2**^	**42.96**
		4	245	11 (9–14)^2^	2.02^0,1,2^	2.41^0,1,2^	42.48
	Alagramam	0	6903	12 (9–21)^1,2,3,4^	15.46^1,2,3,4^	3.38^3,4^	-
		1	2906	11 (9–22)^0,2,3,4^	20.44^0,3,4^	3.37^3,4^	0.30
		2	2148	13 (10–24)^0,1,3,4^	22.38^0,3,4^	3.45^3,4^	-2.07
		3	1966	12 (10–19)^0,1,2,4^	11.11^0,1,2,4^	2.87^0,1,2^	15.09
		**4**	**2790**	**11 (9–17)**^**0,1,2,3**^	**7.37**^**0,1,2,3**^	**2.80**^**0,1,2**^	**17.16**
	Peneng	0	4195	12 (7–26)^2,3,4^	32.02^2,3,4^	4.26^2,3,4^	-
		1	3772	12 (6–26)^2,3,4^	30.86^2,3,4^	4.24^2,3,4^	0.47
		2	1531	10 (7–13)^0,1^	2.22^0,1^	2.53^0,1^	40.61
		**3**	**1581**	**10 (8–13)**^**0,1**^	**2.19**^**0,1**^	**2.53**^**0.1**^	**40.61**
		4	1655	10 (7–13)^0,1^	2.33^0,1^	2.56^0,1^	39.91
TRANSFIL	Kirare	0	6866	13 (7–43)^1,2,3,4^	81.78^1,2,3,4^	4.66^1,2,3,4^	-
		1	17625	11 (7–27)^0,2^	32.62^0.2,3,4^	4.00^0,2,3,4^	14.16
		2	6414	13 (10–26)^0,1,3,4^	22.26^0,1,3,4^	3.50^0,1,3,4^	24.89
		3	2108	11 (9–15)^2^	3.19^0,1,2,4^	2.56^0,1,2,4^	45.06
		**4**	**5405**	**11 (9–15)**^**2**^	**2.83**^**0,1,2,3**^	**2.54**^**0.1,2,3**^	**45.49**
	Alagramam	0	9666	15 (9–42)^2,3,4^	72.86^1,2,3,4^	4.60^1,2,3,4^	-
		1	9109	15 (9–50)^2,3,4^	155.57^0,3,4^	4.52^0,2,3,4^	1.74
		2	5555	18 (11–50)^0,1,3,4^	146.86^0,3,4^	4.59^0,1,3,4^	0.22
		**3**	**528**	**12 (11–15)**^**0,1,2**^	**4.46**^**0,1,2,4**^	**2.02**^**0,1,2,4**^	**56.09**
		4	383	11 (10–15)^0,1,2^	5.33^0,1,2,3^	2.46^0,1,2,3^	46.52
	Peneng	0	7014	21 (8–48)^1,2,3,4^	100.37^1,2,3,4^	5.16^1,2,3,4^	-
		1	55425	16 (7–41)^0,2,3,4^	70.43^0,2,3,4^	4.81^0.2,3,4^	6.78
		2	8892	10 (6–22)^0,1,3,4^	15.99^0,1,3,4^	3.77^0,1,3,4^	26.94
		**3**	**7018**	**11 (7–22)**^**0,1,2,4**^	**14.54**^**0,1,2**^	**3.62**^**0,1,2,4**^	**29.84**
		4	13922	11 (7–22)^0,1,2,3^	14.99^0,1,2^	3.70^0,1,2,3^	28.29

The lowest entropy scenario for each site is bolded and shaded grey. Additional scenarios shaded grey are not significantly different from the lowest entropy scenario.

^#^Scenario 0: model-only; Scenario 1: baseline data; Scenario 2: baseline + post-MDA 3 data; Scenario 3: baseline + post-MDA 3 + post-MDA 5 data; Scenario 4: baseline + post-MDA 5 data

*For each pair of scenarios, pairwise F-tests for equality of variance were performed to compare the weighted variance, differential Shannon entropy tests were performed to compare the entropy, and Kruskal-Wallis multiple comparison tests were performed to compare medians. Pairwise significance is represented by reporting those scenarios which are statistically significantly different from each other by numbers (0–4) as superscripts. For example, the weighted variance for scenario 0 for Kirare has the superscript numbers (1–4) to indicate that the weighted variance for scenario 0 is significantly different from the weighted variance for scenarios 1–4. Significance was determined using the Benjamini-Hochberg procedure for controlling the false discovery rate (q = 0.05) in all pairwise statistical tests.

^+^information gained by each data stream (scenario 1–4) are presented in comparison to the information contained in the model-only simulation (scenario 0)

### Inter-and pooled model performance

**Fig 2:**
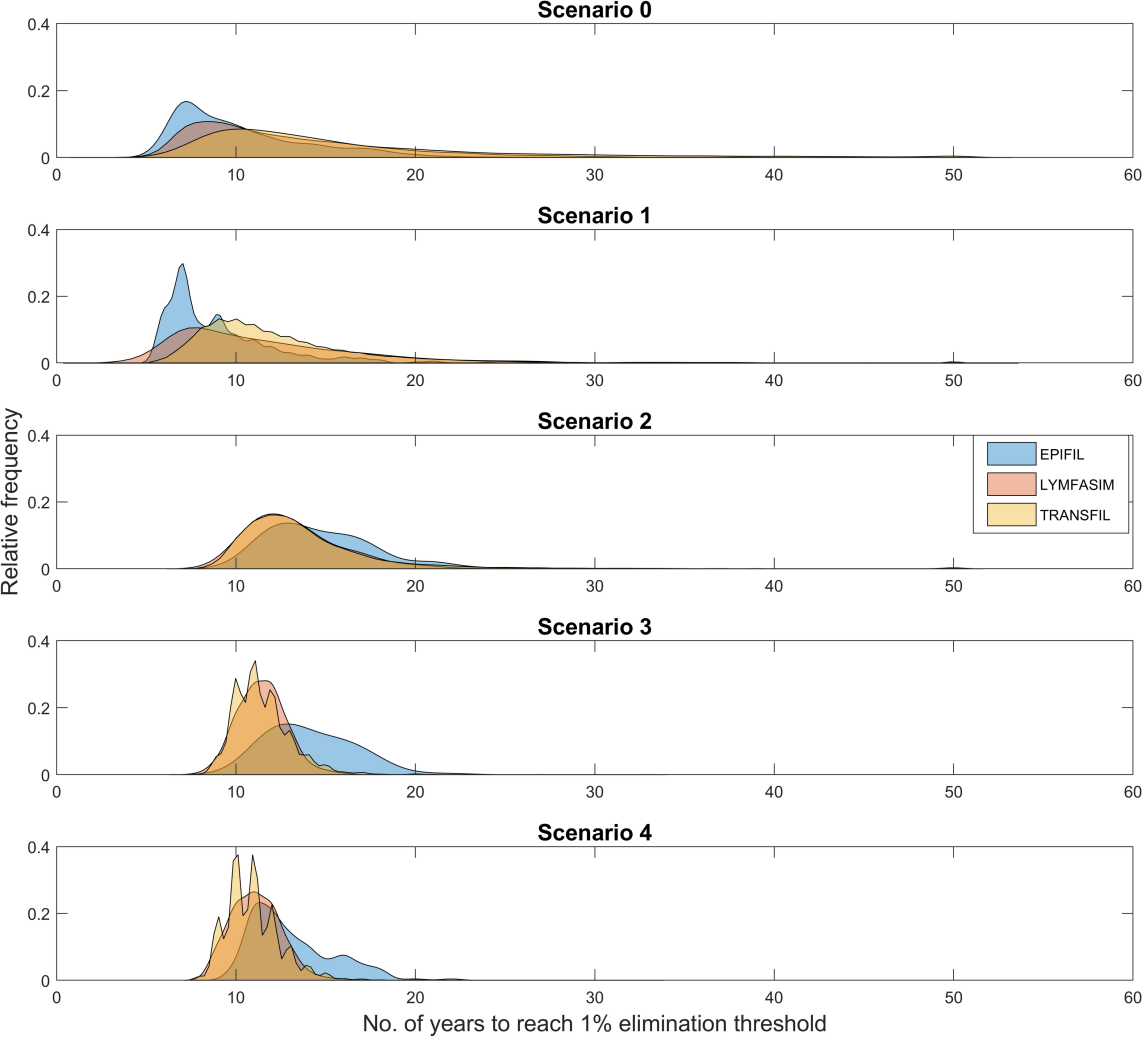
Comparison of the distributions of predicted timelines to LF elimination from the three models for Kirare, Tanzania. This visual comparison shows that the predictions coming from the model-only simulations (scenario 0) have the widest spread in their distributions for all three models compared to model predictions obtained via constraining using subsequent data scenarios. Pairwise Kolmogorov-Smirnov tests for equal distributions were performed on the results from each model to evaluate whether updating the models with sequential data changed the distribution of predictions. Significance was determined using the Benjamini-Hochberg procedure for controlling the false discovery rate (q = 0.05). Apart from scenarios 2 and 3 for EPIFIL and scenarios 3 and 4 for LYMFASIM, all distributions were significantly different from one another (see S2 Supplementary Information for results from the villages of Alagramam and Peneng).

The change in the distributions of predicted timelines to LF elimination without and with model constraining by the different longitudinal data streams is illustrated in Fig 2 for the Kirare site (see S2 Supplementary Information for results obtained for the other two study villages investigated here). The results illustrate that both the location and length of the tail of the prediction distributions can change as models are constrained with increasing lengths of longitudinal data, with inclusion of post-MDA 5 mf survey data consistently producing a narrower or sharper range of predictions compared to when this survey point is excluded.

**Fig 3:**
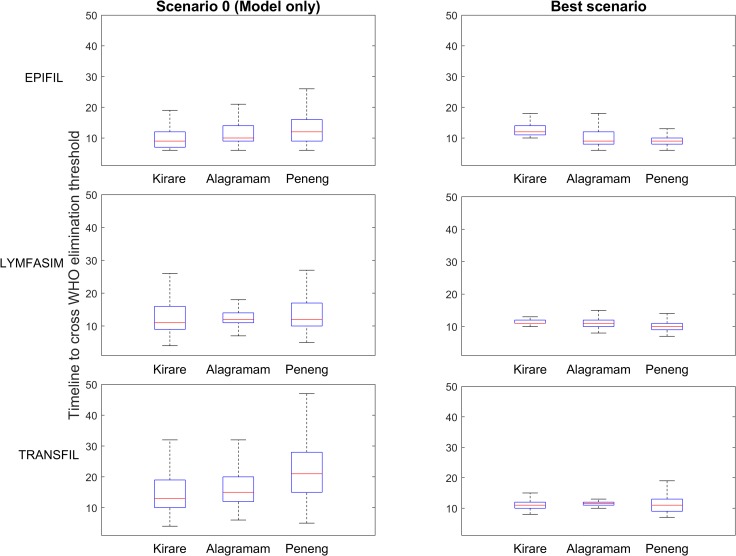
Comparison of model-predicted timelines from model-only simulations and the lowest entropy simulations in each site. The boxplots show that by calibrating the models to data streams, more precise predictions are able to be made regarding timelines to achieve 1% mf prevalence across all models and sites. The results of pairwise *F*-tests for variance, performed to compare the weighted variance in timelines to achieve 1% mf prevalence between model-only simulations (scenario 0) and the lowest entropy simulations (best scenario) (see Table 2), show that the predictions for the best scenarios are significantly different from the predictions for the model-only simulations. Significance was determined using the Benjamini-Hochberg procedure for controlling the false discovery rate (q = 0.05). For EPIFIL, LYMFASIM and TRANSFIL, the best scenarios are scenarios 4, 3, and 4 for Kirare, scenarios 4, 4, and 3 for Alagramam, and scenarios 3, 3, and 3 for Peneng, respectively.

Fig 3 compares the uncertainty in predictions of timelines to achieve elimination made by each of the three models without (scenario 0) and via their constraining by the data streams providing the lowest prediction entropy for each of the models per site. Note that variations in scenario 0 predictions among the three models directly reflect the different model structures, parameterizations, and the presence (or absence) of stochastic elements. The boxplots in the figure, however, show that for all three sites and models, calibration of each model by data greatly reduces the uncertainty in predictions of the years of annual MDA required to eliminate LF compared to model-only predictions, with the data streams producing the lowest entropy for simulations in each site significantly improving the precision of these predictions (Table 2). This gain in precision, and thus the information gained using these data streams, is, as expected, greater for the stochastic LYMFASIM and TRANSFIL models compared to the deterministic EPIFIL model. Note also that even though the ranges in predictions of the annual MDA years required to eliminate LF by the data streams providing the lowest prediction entropy differed statistically between the three models, the values overlapped markedly (e.g. for Kirare the ranges are 10–18, 9–14, 9–15 for EPIFIL, LYMFASIM and TRANSFIL respectively), suggesting the occurrence of a similar constraining of predictive behaviour among the three models.

**Fig 4:**
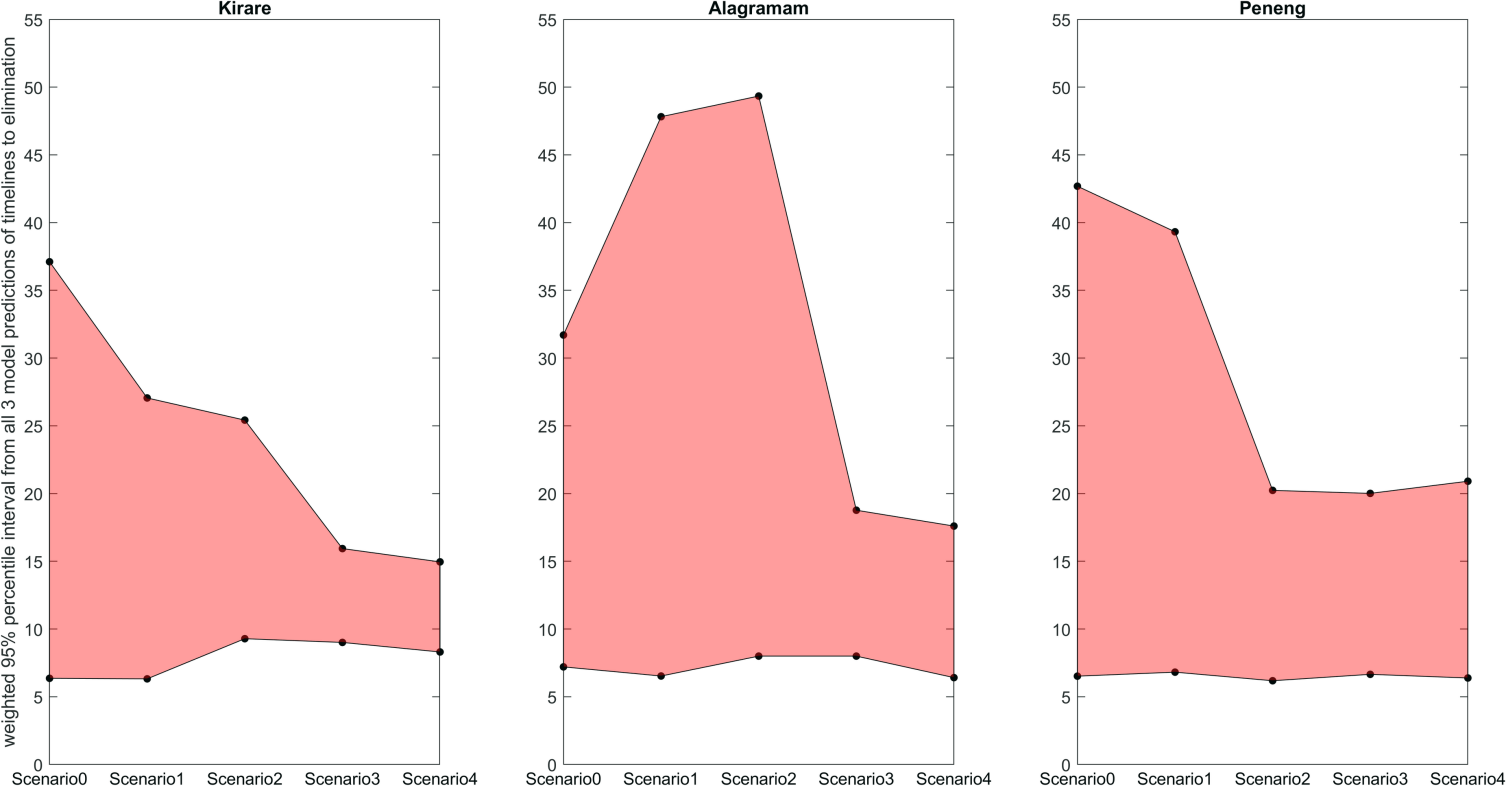
Pooled predictions of the timelines to reach 1% mf from three LF models. The shaded regions show the weighted 95% percentile interval from the composite predictions of all three models of the timelines required to cross the WHO 1% elimination threshold for all five scenarios. The black dots indicate upper and lower bounds (weighted 2.5^th^ and 97.5^th^ percentiles) of the composite predictions from all three models for each scenario. The range of predictions is tightest when the models were constrained with data from scenarios 3 and 4.

To investigate this potential for a differential model effect, we further pooled the predictions from all three models for all the data scenarios and evaluated the value of each investigated data stream for improving their combined predictive ability. The weighted 95% percentile intervals from the pooled predictions were used for carrying out this assessment. The results are depicted in Fig 4 and indicate that, as for the individual model predictions, uncertainty in the collective predictions by the three LF models for the required number of years to eliminate LF using annual MDA in each site may be reduced by model calibration to data, with the longitudinal mf prevalence data collected during the later monitoring periods (scenarios 3 and 4) contributing most to improving the multi-model predictions for each site.

### Parameter constraints and interactions

**Fig 5:**
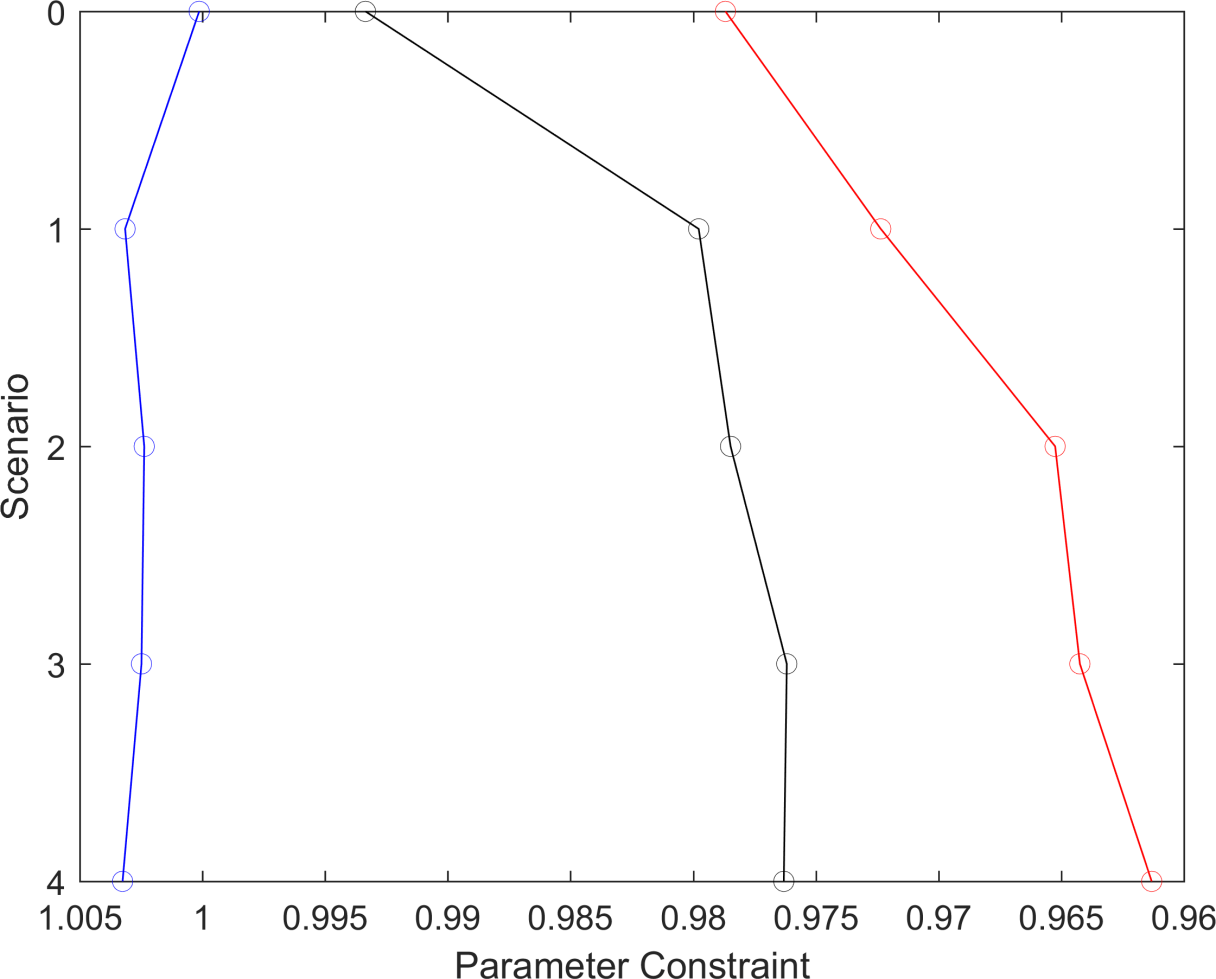
Parameter constraint achieved through the coupling of EPIFIL with data. Overall parameter constraint was measured as the ratio of the mean standard deviation of the fitted parameter distributions to that of the prior parameter distributions. Values < 1 indicate that the fitted parameter space was constrained compared to the prior parameter space. The results show that the fitted parameter space for Kirare and Peneng was more constrained by calibrating the model to data compared to the model-only scenario, but this was not the case for Alagramam.

We attempted to investigate if model uncertainty in predictions by the use of longitudinal data was a direct function of parameter constraining by the addition of data. Given the similarity in outcomes of each model, we remark on the results from the fits of the technically easier to run EPIFIL model to evaluate this possibility here. The assessment of the parameter space constraint achieved through the inclusion of data was made by determining if the fitted parameter distributions for the model became reduced in comparison with priors as data streams were added to the system [33]. The exercise showed that the size of the estimated parameter distributions reduced with addition of data, with even scenario 1 data producing reductions for Kirare and Peneng (Fig 5). In the case of Alagramam, however, there was very little, if any, constraint in the fitted parameter space compared to the prior parameter space. This result, together with the fact that even using all the data in Kirare and Peneng produced up to only between 2.5 to 5% reductions in fitted parameter distributions when compared to the priors, indicate that the observed model prediction uncertainty in this study may be due to other complex factors connected with model parameterization. Table 3 provides the results of an analysis of pairwise parameter correlations of the selected best-fitting models for data scenario 1 compared to those selected by the data stream that gave the best reduction in EPIFIL prediction uncertainty for Alagramam (scenario 3). These results show that while the parameter space was not being constrained with the addition of more data, the pattern of parameter correlations changed in a complex manner between the two constraining data sets. For example, although the number of significantly correlated parameters did not differ, the magnitude and direction of parameter correlations were shown to change between the two data scenarios (Table 3). The corresponding results for Kirare and Peneng are shown in S3 Supplementary Information, and indicate that a broadly similar pattern of changes in parameter associations also occurred as a result of model calibration to the sequential data measured from those sites. This suggests that this outcome may constitute a general phenomenon at least with regards to the sequential constraining of EPIFIL using longitudinal mf prevalence data. An intriguing finding (from all three data settings) is that the most sensitive parameters in this regard, i.e. with respect to altered strengths in pairwise parameter correlations, may be those representing the relationship of various components of host immunity with different transmission processes, including with adult worm mortality, rates of production and survival of mf, larval development rates in the mosquito vector and infection aggregation (Table 3). This suggests that, as more constraining data are added, changes in the multidimensional parameter relationships related to host immunity could contribute to the sequential reductions in the LF model predictive uncertainty observed in this study.

**Table 3:** Spearman parameter correlations for scenarios 1 (lower left triangle) and 3 (upper right triangle) for Alagramam, India.

	λ	α	k_0_	k_Lin_	κ	r	σ	Ψ_1_	Ψ_2_	μ	γ	g	c	H_Lin_	I_c_	S_c_	τ	δ
λ		**-0.068**	-0.008	**-0.025**	0.002	**-0.041**	0.012	**-0.031**	**-0.095**	0.005	**0.022**	**-0.030**	<0.001	0.012	0.006	-0.018	-0.016	**-0.026**
α	**-0.071**		-0.005	**-0.191**	-0.003	**-0.157**	**0.095**	**-0.150**	**-0.453**	**0.083**	**0.048**	**-0.079**	**0.031**	**0.029**	0.005	-0.016	0.013	**-0.045**
k_0_	-0.001	**-0.016**		0.002	**0.021**	0.007	-0.008	-0.007	0.007	0.011	-0.001	-0.018	<0.001	0.014	-0.004	0.013	-0.005	-0.018
k_Lin_	**-0.022**	**-0.186**	0.006		0.003	**-0.085**	**0.034**	**-0.045**	**-0.150**	**0.027**	**0.032**	**-0.030**	**0.027**	0.013	**-0.023**	-0.001	**0.033**	**-0.055**
κ	-0.005	-0.004	0.011	0.001		**-0.022**	-0.001	-0.012	**-0.019**	0.007	0.004	-0.012	-0.011	0.002	**-0.023**	-0.009	-0.003	0.008
r	**-0.044**	**-0.152**	0.008	**-0.079**	**-0.017**		**0.060**	**-0.067**	**-0.240**	**0.037**	**0.035**	-0.013	0.008	0.017	-0.012	0.005	-0.003	0.005
σ	0.012	**0.090**	-0.002	**0.040**	0.011	**0.055**		**0.022**	**0.115**	-0.019	-0.010	**0.025**	0.001	0.003	**0.020**	0.017	-0.012	0.012
Ψ_1_	**-0.024**	**-0.143**	-0.012	**-0.049**	-0.011	**-0.075**	0.011		**-0.168**	**0.029**	**0.023**	**-0.028**	0.010	-0.009	-0.013	-0.011	-0.004	-0.015
Ψ_2_	**-0.094**	**-0.454**	0.012	**-0.151**	-0.009	**-0.248**	**0.125**	**-0.170**		**0.102**	**0.072**	**-0.104**	0.016	**0.033**	**-0.034**	**-0.024**	**0.049**	**-0.060**
μ	0.008	**0.070**	0.006	**0.030**	0.009	**0.043**	**-0.026**	**0.029**	**0.104**		**-0.025**	0.009	-0.001	0.012	0.001	-0.005	0.019	0.016
γ	**0.019**	**0.055**	-0.004	**0.034**	0.005	**0.030**	-0.014	**0.024**	**0.072**	-0.015		**0.023**	**-0.019**	-0.010	0.003	0.002	**0.020**	0.010
g	**-0.028**	**-0.078**	-0.010	**-0.031**	-0.015	**-0.016**	**0.026**	**-0.024**	**-0.101**	**0.016**	0.014		0.006	0.007	**-0.026**	0.010	0.006	-0.013
c	-0.001	**0.026**	<0.001	**0.019**	-0.002	0.012	0.004	0.006	**0.023**	-0.007	**-0.023**	0.004		0.003	**0.031**	-0.003	-0.010	0.007
H_Lin_	0.009	**0.025**	0.009	0.011	0.015	0.009	0.008	-0.008	**0.050**	0.003	-0.004	0.002	0.011		0.015	-0.008	0.006	0.007
I_c_	0.010	<0.001	-0.010	**-0.020**	-0.014	**-0.016**	**0.019**	-0.015	**-0.028**	0.010	0.002	-0.014	**0.016**	0.003		**-0.061**	-0.005	**-0.026**
S_c_	-0.013	-0.002	0.007	-0.005	-0.008	0.004	0.003	-0.005	**-0.029**	0.001	0.005	-0.005	0.003	-0.008	**-0.057**		-0.015	-0.011
τ	-0.003	**0.019**	-0.002	**0.029**	-0.004	-0.001	0.001	-0.005	**0.043**	0.001	**0.023**	0.009	-0.005	0.009	-0.004	-0.009		0.003
δ	-0.014	**-0.048**	-0.009	**-0.051**	0.003	0.001	0.003	**-0.016**	**-0.060**	0.007	0.010	-0.010	0.011	0.005	**-0.020**	**-0.017**	0.001	

Cell formatting reflects significant correlations (bold text), correlation coefficient sign changes between the two scenarios (bold bordered cells), and more than two fold magnitude changes in the correlation coefficients between the two scenarios (blue cells indicate the correlation was stronger in scenario 1 and red cells indicate the correlation was stronger in scenario 3).

### Contributions of MDA coverage and length of monitoring data to model uncertainty

**Fig 6:**
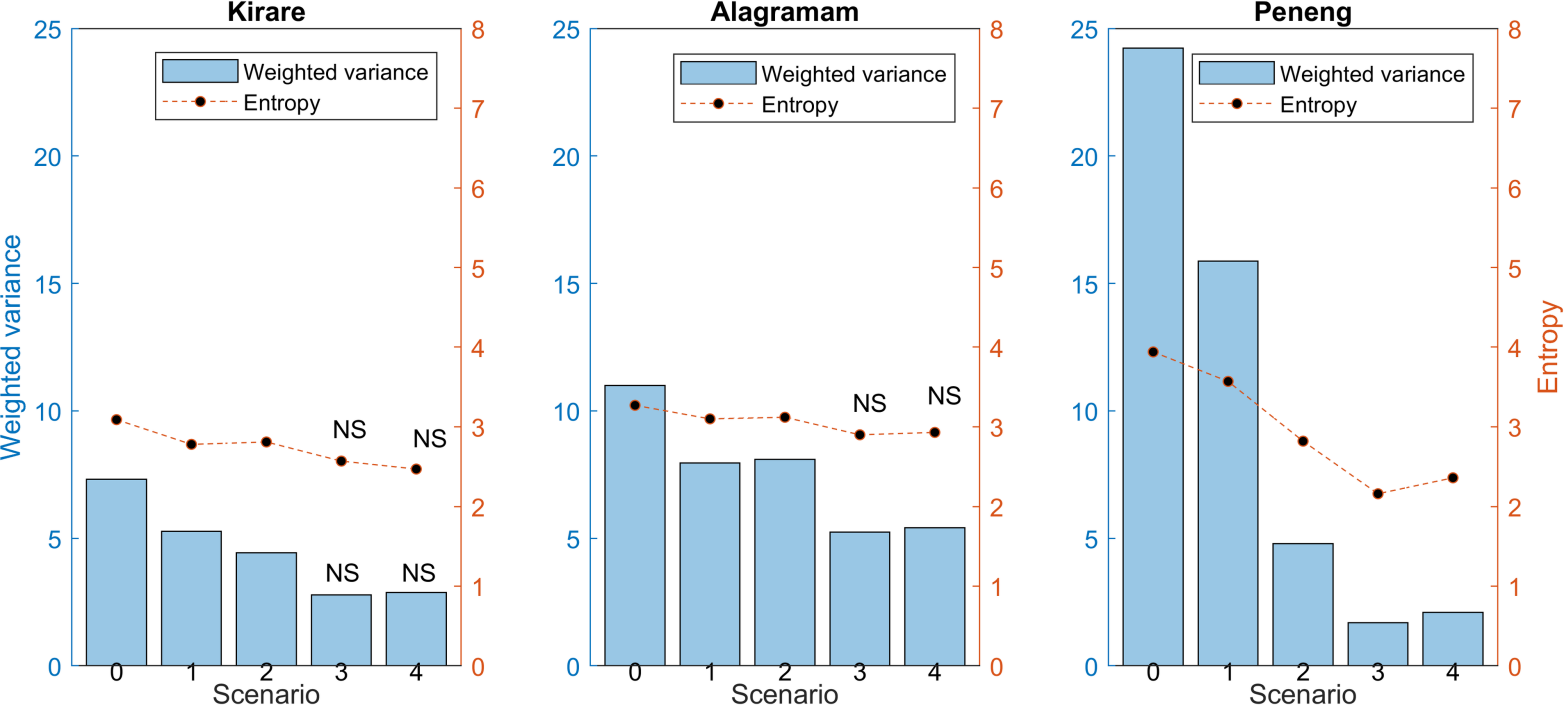
Weighted variance and entropy values of EPIFIL predictions of LF elimination timelines using optimal MDA coverage in each study site. For all sites, either scenario 3 or 4 had the lowest entropies, and scenario 4 was not significantly different from scenario 3 for Kirare and Alagramam. These results were not statistically different from the results given 65% coverage (see Table 2), suggesting that the data stream associated with the lowest entropy is robust to changes in the interventions simulated. Scenarios where the weighted variance or entropy were not significantly different from the lowest entropy scenario are noted with the abbreviation NS. Significance was determined using the Benjamini-Hochberg procedure for controlling the false discovery rate (q = 0.05).

The LF elimination timeline predictions used above were based on modelling the impacts of annual MDA given the reported coverages in each site followed by an assumed standard coverage for making longer term predictions (see Methods). This raises the question as to whether the differences detected in the case of the best constraining data stream between the present study sites and between models (Table 2) could be a function of the simulated MDA coverages in each site. To investigate this possibility, we used EPIFIL to model the outcome of changing the assumed MDA coverage in each site on the corresponding entropy and information gain trends in elimination predictions made from the models calibrated to each of the site-specific data scenarios/streams investigated here. The results of increasing the assumed coverage of MDA to 80% for each site are shown in Fig 6 and indicate that the choice of MDA coverage in this study are unlikely to have significantly influenced the conclusion made above that the best performing data streams for reducing model uncertainty for predicting LF elimination pertains to data scenarios 3 and 4. However, while the model-predicted timelines to achieve the 1% mf prevalence threshold using the observed MDA coverage followed by 80% MDA coverage showed that the data stream which most reduced uncertainty did not change from the impact of using the observed MDA coverage followed by 65% MDA coverage modelled for Kirare and Peneng (Table 2, Fig 6), this was not the case for Alagramam, where data from scenario 3 with a 80% coverage resulted in the greatest reduction in entropy compared to the original results using 65% coverage which indicated that scenario 4 data performed best (Table 2, Fig 6). Notably, though, the entropy values of predictions using the data scenario 3 and 4 constraints were not statistically different for this site (*p*-value < 0.05) (Fig 6).

**Table 4:** Predictions of timelines to achieve 1% mf in Villupuram district, India, considering extended post-MDA data.

*Scenario*^*#*^	*No. of accepted models*	*Median no. of years* *(2.5^th^-97.5^th^ percentiles)* ^(significance^*)	*Weighted variance* ^(significance^*)	*Entropy* ^(significance^*)	*Relative information gained by data (%)*^*+*^
*0*	15419	10 (7–23)^1,2,3,4,5,6^	19.62^1,2,3,4,5,6^	3.69^1,2,3,4,5,6^	-
*1*	16352	7 (3–17)^0,2,3,5,6^	12.42^0,2,3,4,5,6^	3.47^0,2,3,4,5,6^	5.96
*2*	11581	8 (6–18)^0,1,4^	10.75^0,1,3,5,6^	3.32^0,1,3,4,5,6^	10.03
*3*	11381	8 (6–16)^0,1,4^	8.92^0,1,2,4^	3.23^0,1,2,4^	12.47
*4*	16152	7 (3–16)^0,2,3,5,6^	10.82^0,1,3,5,6^	3.40^0,1,2,3,5,6^	7.86
*5*	11381	8 (6–16)^0,1,4^	8.92^0,1,2,4^	3.23^0,1,2,4^	12.47
***6***	**11369**	**8 (6–16)**^**0,1,4**^	**8.80**^**0,1,2,4**^	**3.22**^**0,1,2,4**^	**12.74**

The lowest entropy scenario for each site is bolded and shaded grey. Additional scenarios shaded grey are not significantly different from the lowest entropy scenario.

^#^Scenario 0–4 are as previously defined; Scenario 5: Baseline + post-MDA 3 + post-MDA 5 + post-MDA 7 data; Scenario 6: Baseline + post-MDA 3 + post-MDA 5 + post-MDA 7 + post-MDA 9 data

*For each pair of scenarios, pairwise F-tests for equality of variance were performed to compare the weighted variance, differential Shannon entropy tests were performed to compare the entropy, and Kruskal-Wallis multiple comparison tests were performed to compare the medians. Pairwise significance is represented by reporting those scenarios which are statistically significantly different from each other by numbers (0–4) as superscripts. For example, the weighted variance for scenario 0 has the superscript numbers (1–6) to indicate that the weighted variance for scenario 0 is significantly different from the weighted variance for scenarios 1–6. Significance was determined using the Benjamini-Hochberg procedure for controlling the false discovery rate (q = 0.05) in all pairwise statistical tests.

^+^information gained by each data stream (scenario1-6) are presented in comparison to the information contained in the model-only simulation (scenario 0)

EPIFIL was also used to expand the number of calibration scenarios using a dataset with longer term post-MDA data from Villupuram district, India. This dataset contained two addition data streams: scenario 5 which included baseline, post-MDA 3, post-MDA 5, and post-MDA 7 mf data, and scenario 6, which included baseline, post-MDA 3, post-MDA 5, post-MDA 7, and post-MDA 9 mf data. Scenario 6 thus contained the most post-MDA data and was demonstrated to be the most effective for reducing model uncertainty, but this effect was not statistically significantly different from the reductions produced by assimilating data contained in scenarios 3 and 5 (Table 4). The inclusion of more data than are considered in scenario 3 therefore did not result in any significant additional reduction in model uncertainty.

### Assessing prediction accuracy

EPIFIL was used to evaluate the accuracy of the data-driven predictions of the timelines required to meet the goal of LF elimination based on breaching the WHO-set target of 1% mf prevalence. This analysis was performed by using the longitudinal pre and post-infection and MDA data reported for the Nigerian site, Dokan Tofa, where elimination was achieved according to WHO recommended criteria after seven rounds of MDA (Table 5). The data from this site comprised information on the ABR and dominant mosquito genus, as well as details of the MDA intervention carried out, including the relevant drug regimen applied, time and population coverage of MDA, and outcomes from the mf prevalence surveys conducted at baseline and at multiple time points during MDA [60]. The results of model predictions of the timelines to reach below 1% mf prevalence as a result of sequential fitting to the mf prevalence data from this site pertaining to scenarios 0–4 (as defined above) are shown in Table 6. Note that in the post MDA 3, 5 and 7 surveys, as no LF positive individuals were detected among the sample populations, we used a one-sided 95% Clopper-Pearson interval to determine the expected upper one-sided 95% confidence limits for these sequentially observed zero infection values using the “Rule of Three” approximation after *K* empty samples formula [61]. The results show that model constraining by scenario 2, which includes baseline and post-MDA 3 data, and scenario 3, which includes baseline, post-MDA 3, and post-MDA 5 data, resulted in both the least entropy values and the shortest predicted times, i.e., from as low as 2 to as high as 7 years, required for achieving LF elimination in this site (Table 6). The data in Table 5 show that the first instance the calculated one-sided upper 95% confidence limit in this setting fell below 1% mf prevalence also occurred post MDA 7 (i.e after 7 years of MDA). This is a significant result, and indicates that apart from being able to reduce prediction uncertainty, the best data-constrained models are also able to more accurately predict the maximal time (7 years) by which LF elimination occurred in this site.

**Table 5:** Annual mf prevalence survey and MDA data for Dokan Tofa, Nigeria.

*Village*	Dokan Tofa			
Regimen (efficacy*)	IVM+ALB (99/9)			
*Mosquito genus*	Anopheles			
*ABR*	300–5000			
	Year	Mf Prev (Pos. no./ no. sampled)	Upper limit of 95% CI of Mf Prev	Total population MDA cov
***Pre-treatment***	2003	5% (21/419)	7.1%	74.9%
***Post MDA 1***	2004	NA	NA	76.7%
***Post MDA 2***	2005	3% (7/236)	5.4%	67.4%
***Post MDA 3***	2006	0% (0/132)	2.2%	77.6%
***Post MDA 5***	2008	0% (0/158)	1.03%	78.3%
***Post MDA 7***	2010	0% (0/119)	0.73%	

Years shaded in grey indicate data used to constrain the model.

* Drug regimen efficacy given as % mf killed instantaneously/number of months of reduced worm fecundity

**Table 6:** EPIFIL predictions of timelines to achieve 1% mf prevalence in Dokan Tofa, Nigeria.

*Site*	*Scenario^*#*^*	*No. of accepted models*	*Median no. of years* (2.5^th^-97.5^th^ percentiles) ^(significance^*^)^	*Weighted variance* ^(significance^*^)^	*Entropy* ^(significance^*^)^	*Relative* *information* *gained by data (%)*^*+*^
*Dokan Tofa*	0	3007	3 (2–10)	2.41^1,2,3,4^	2.55^1,2,3,4^	-
	1	2046	3 (2–8)	2.40^0,2,3^	2.45^0,2,3^	0.41
	2	2007	3 (2–7)	2.07^0,1,4^	2.35^0,1,4^	0.85
	**3**	**2007**	**3 (2–7)**	**2.07**^**0,1,4**^	**2.35**^**0,1,4**^	**0.85**
	4	2046	3 (2–8)	2.40^0,2,3^	2.45^0,2,3^	0.41

The lowest entropy scenario for each site is bolded and shaded grey. Additional scenarios shaded grey are not significantly different from the lowest entropy scenario.

^#^Scenario 0: model-only; Scenario 1: baseline data; Scenario 2: baseline + post-MDA 3 data; Scenario 3: baseline + post-MDA 3 + post-MDA 5 data; Scenario 4: baseline + post-MDA 5 data

*For each pair of scenarios, pairwise *F*-tests for equality of variance were performed to compare the weighted variance, differential Shannon entropy tests were performed to compare the entropy, and Kruskal-Wallis multiple comparison tests were performed to compare medians. Pairwise significance is represented by reporting those scenarios which are statistically significantly different from each other by numbers (0–4) as superscripts. For example, the weighted variance for scenario 0 for Kirare has the superscript numbers (1–4) to indicate that the weighted variance for scenario 0 is significantly different from the weighted variance for scenarios 1–4. Significance was determined using the Benjamini-Hochberg procedure for controlling the false discovery rate (q = 0.05) in all pairwise statistical tests.

^+^information gained by each data stream (scenario 1–4) are presented in comparison to the information contained in the model-only simulation (scenario 0)

## Discussion

Our major goal in this study was to compare the reliability of forecasts of timelines required for achieving parasite elimination made by generic LF models versus models constrained by sequential mf prevalence surveillance data obtained from field sites undergoing MDA. A secondary aim was to evaluate the relative value of data obtained at each of the sampling time points proposed by the WHO for monitoring the effects of LF interventions in informing these model predictions. This assessment allowed us to investigate the role of these data for learning system dynamics and measure their value for guiding the design of surveillance programmes in order to support better predictions of the outcomes of applied interventions. Fundamentally, however, this work addresses the question of how best to use predictive parasite transmission models for guiding management decision making, i.e. whether this should be based on the use of ideal models which incorporate generalized parameter values or on models with parameters informed by local data [10]. If we find that data-informed models can reduce prediction uncertainty significantly compared to the use of theoretical models unconstrained by data, then it is clear that to be useful for management decision making we require the application of model-data assimilation frameworks that can effectively incorporate information from appropriate data into models for producing reliable intervention projections. Antithetically, such a finding implies that using unconstrained ideal models in these circumstances will provide only approximate predictions characterized by a degree of uncertainty that might be too large to be useful for reliable decision making [14, 33, 62].

Here, we have used three state-of-the-art LF models calibrated to longitudinal human mf prevalence data obtained from three representative LF study sites to carry out a systematic analysis of these questions in parasite intervention modelling (see also Walker et al [63] for a recent study highlighting the importance of using longitudinal sentinel site data for improving the prediction performances of the closely-related onchocerciasis models). Further, by iteratively testing the reduction in the uncertainty of the projections of timelines required to achieve LF elimination in a site made by the models matching each observed data point, we have also quantified the relative values of temporal data streams, including assessing optimal record lengths, for informing the current LF models. Our results provide important insights as to how best to use process models for understanding and generating predictions of parasite dynamics. They also highlight how site-specific longitudinal surveillance data coupled with models can be useful for providing information about system dynamics and hence for improving predictions of relevance to management decision-making.

The first result of major importance from our work is that models informed by data can significantly reduce predictive uncertainty and hence improve performance of the present LF models for guiding policy and management decision-making. Our results show that these improvements in predictive precision were consistent between the three models and across all three of our study sites, and can be very substantive with up to as much as 92% to 96% reductions in prediction variance obtained by the best data-constrained models in a site compared to the use of model-only predictions (Table 2). The practical policy implications of this finding can also be gleaned from appraising the actual numerical ranges in the predictions made by each individual model for each of the modelling scenarios investigated here. In the case of EPIFIL, the best data-informed model (scenario 3 in Peneng) gave an elimination prediction range of 7–12 years, while the corresponding model-only predictions for this site indicated a need for between 6–29 years of annual MDA (Table 2). These gains in information from using data to inform model parameters and hence predictions were even larger for the two stochastic models investigated here, viz. LYMFASIM and TRANSFIL, where ranges as wide as 7–28 years predicted by model-only scenarios were reduced to 9–14 years for the best data-informed models in the case of LYMFASIM for Kirare village, and from as broad as 8–48 years to 7–22 years respectively in the case of TRANSFIL for Peneng (Table 2). These results unequivocally indicate that if parasite transmission models are used unconstrained by data, i.e. based on general parameter values uninformed by local data, it would lead to the making of predictions that would be marked by uncertainties that are likely to be far too large to be meaningful for practical policy making. If managers are risk averse, this outcome will also mean their need to plan interventions for substantially much longer than necessary, with major implications for the ultimate cost of the programme. Note also that although statistically significant changes in the median years of MDA required to achieve LF elimination were observed for the best data-informed models for all the three LF model types in each site, these were relatively small compared to the large reductions seen in each model’s predictive uncertainly (Table 2, Fig 3). This result highlights that the major gains from constraining the present models by data lies in improving their predictive certainty rather than in advancing their average behaviour. However, our preliminary analysis of model predictive accuracy suggests that the best data-constrained models may also be able to generate more accurate predictions of the impact of control (Table 6), indicating that, apart from simply reducing predictive uncertainty, such models could additionally have improved capability for producing more reliable predictions of the outcomes of interventions carried out in a setting.

The iterative testing of the reduction in forecast uncertainty using mf surveillance data measured at time points proposed by the WHO (to support assessment of whether the threshold of 1% mf prevalence has been reached before implementation units can move to post-treatment surveillance [44]) has provided further insights into the relative value of these data for improving the predictive performance of each of the present LF models. Our critical finding here is that parameter uncertainty in all three LF models was similarly reduced by the assimilation of a few additional longitudinal data records (Table 2). In particular, we show that data streams comprising baseline + post-MDA 3 + post-MDA 5 (scenario 3) and those comprising baseline + post-MDA 5 data (scenario 4) best reduced parameter-based uncertainty in model projections of the impact of MDAs carried out in each study site irrespective of the models used. Although preliminary, a potential key finding is that the use of longer-term data additional to the data measured at the WHO proposed monitoring time points did not lead to a significant further reduction in parameter uncertainty (Table 4). Also, the finding that the WHO data scenarios 3 and 4 were adequate for constraining the present LF models appears not to be an artefact of variations in the MDA coverages observed between the three study sites (Fig 6). These results suggest that up to 5 years of post-MDA mf prevalence data are sufficient to constrain model predictions of the impact of LF interventions at a time scale that can go up to as high as 7 to 22 years depending on the site and model, and that precision may not improve any further if more new data are added (Table 2, Table 4). Given that the WHO post-MDA LF infection monitoring protocol was developed for the purpose solely focussed on supporting the meeting of set targets (e.g. the 1% mf prevalence threshold) and not on *a priori* hypotheses regarding how surveillance data could be used also to understand the evolution and hence prediction of the dynamical parasitic system in response to management action, our results are entirely fortuitous with respect to the value of the current LF monitoring data for learning about the LF system and its extinction dynamics in different settings [31]. They do, nonetheless, hint at the value that coupling models to data may offer to inform general theory for guiding the collection and use of monitoring data in parasite surveillance programmes in a manner that could help extract maximal information about the underlying parasite system of interest.

Our assessment of whether the incremental increase in model predictive performance observed as a result of assimilating longitudinal data may be due to parameter constraining by the addition of data has shed intriguing new light on the impact that qualitative changes in dynamical system behaviour may have on parameter estimates and structure, and hence on the nature of the future projections of system change we can make from models. Our major finding in this regard is that even though the parameter space itself may not be overly constrained by the best data stream (scenario 3 in this case for Alagramam village), the magnitude and direction of parameter correlations, particularly those representing the relationship of different components of host immunity with various transmission processes, changed markedly between the shorter (scenario 1) and seemingly optimal data streams (scenario 3). This qualitative change in system behaviour induced by alteration in parameter interactions in response to perturbations has been shown to represent a characteristic feature of complex adaptive ecological systems, particularly when these systems approach a critical boundary [64–66]. This underscores yet another important reason to incorporate parameter information from data for generating sound system forecasts [67]. The finding that additional data beyond 5 years post-MDA did not appear to significantly improve model predictive performance in this regard suggests that pronounced change in LF parameter interactions in response to MDA interventions may occur generally around this time point for this parasitic disease, and that once in this parameter regime further change appears to be unlikely. This is an interesting finding, which not only indicates that coupling models to at least 5 years post-MDA will allow detection of the boundaries delimiting the primary LF parameter regions with different qualitative behaviour, but also that the current WHO monitoring protocol might be sufficient to allow this discovery of system change.

Although our principal focus in this study was in investigating the value of longitudinal data for informing the predictive performance of the current LF models, the results presented here have also underscored the existence of significant spatial heterogeneity in the dynamics of parasite extinction between the present sites (Table 2, Fig 3). In line with our previous findings, this observed conditional dependency of systems dynamics on local transmission conditions means that timelines or durations of interventions required to break LF transmission (as depicted in Table 2) will also vary from site to site even under similar control conditions [3–5, 21]. As we indicated before, this outcome implies that we vitally require the application of models to detailed spatio-temporal infection surveillance data, such as that exemplified by the data collected by countries in sentinel sites as part of their WHO-directed monitoring and evaluation activities, if we are to use the present models to make more reliable intervention predictions to drive policy and management decisions (particularly with respect to the durations of interventions required, need for switching to more intensified or new MDA regimens, and need for enhanced supplementary vector control) in a given endemic setting [64]. As we have previously pointed out, the development of such spatially adaptive intervention plans will require the development and use of spatially-explicit data assimilation modelling platforms that can couple geostatistical interpolation of model inputs (eg. ABR and/or sentinel site mf/antigen prevalence data) with discovery of localized models from such data in order to produce the required regional or national intervention forecasts [5].

The estimated parameter and prediction uncertainties presented here are clearly dependent on the model-data fusion methodology and its implementation, and the cost function used to discover the appropriate models for a data stream [20]. While we have attempted to evaluate differences in individual model structures, their computer implementation, and the data assimilation procedures followed (e.g. sequential vs. simultaneous data assimilation), via comparing the collective predictions of the three models versus the predictions provided by each model singly, and show that these factors are unlikely to play a major role in influencing the current results, we indicate that future work must address these issues adequately to improve the initial methods we have employed in this work. Currently, we are examining the development of sequential Bayesian-based multi-model ensemble approaches that will allow better integration of each model’s behaviour as well as better calculation of each model’s transient parameter space at each time a new observation becomes available [30]. This work also involves the development of a method to fuse information from several indicators of infection (e.g. mf, antigenemia, antibody responses [21]) together to achieve a more robust constraining of the present models. As different types of data can act as mutual constraints on a model, we also expect that such multi-indicator model-data fusion methods will additionally address the problem of equifinality, which is known to complicate the parameterization of complex dynamical models [24, 68].

Of course, the ultimate test of the results reported here, viz. that LF models constrained by coupling to year 5 post-MDA data can provide the best predictions of timelines for meeting the 1% mf prevalence threshold in a site, is by carrying out the direct validation of our results against independent observations (as demonstrated by the preliminary validation study carried out here using the Dokan Tofa data (Tables 5 and 6)). We expect that data useful for performing these studies at scale may be available at the sentinel site level in the countries carrying out the current WHO-led monitoring programme. The present results indicate that access to such data, and to post-treatment surveillance data which are beginning to be assembled by many countries, is now a major need if the present LF models are to provide maximal information about parasite system responses to management and thus generate better predictions of system states for use in policy making and in judging management effectiveness in different spatio-temporal settings [24, 31]. Given that previous modelling work has indicated that if the globally fixed WHO-proposed 1% mf prevalence threshold is insufficient to break LF transmission in every setting (and thus conversely leading to significant infection recrudescence [21]), the modelling of such spatio-temporal surveillance data will additionally allow testing if meeting this recommended threshold will indeed result in successfully achieving the interruption of LF transmission everywhere.

## Supporting information

S1 Supplementary Information Lymphatic Filariasis model descriptions. (DOCX)

S2 Supplementary Information Model-predicted timelines to achieve elimination. (DOCX)

S3 Supplementary Information Spearman parameter correlations for Kirare and Peneng. (DOCX)

## References

[1] Michael E. The epidemiology of filariasis control. The Filaria. 2002:59–74.

[2] Michael E, Malecela‐Lazaro MN, Kazura JW. Epidemiological modelling for monitoring and evaluation of lymphatic filariasis control. Advances in parasitology. 2007;65:191–237. 10.1016/S0065-308X(07)65003-9 18063097

[3] Michael E, Malecela-Lazaro MN, Simonsen PE, Pedersen EM, Barker G, Kumar A, et al Mathematical modelling and the control of lymphatic filariasis. The Lancet infectious diseases. 2004;4(4):223–34. 10.1016/S1473-3099(04)00973-9 15050941

[4] Michael E, Singh BK. Heterogeneous dynamics, robustness/fragility trade-offs, and the eradication of the macroparasitic disease, lymphatic filariasis. Bmc Med. 2016;14 10.1186/s12916-016-0557-y PubMed PMID: WOS:000368769400002. 26822124 PMC4731922

[5] Michael E, Singh BK, Mayala BK, Smith ME, Hampton S, Nabrzyski J. Continental-scale, data-driven predictive assessment of eliminating the vector-borne disease, lymphatic filariasis, in sub-Saharan Africa by 2020. Bmc Medicine. 2017;15(1):176 10.1186/s12916-017-0933-2 28950862 PMC5615442

[6] Singh BK, Bockarie MJ, Gambhir M, Siba PM, Tisch DJ, Kazura J, et al Sequential Modelling of the Effects of Mass Drug Treatments on Anopheline-Mediated Lymphatic Filariasis Infection in Papua New Guinea. Plos One. 2013;8(6). doi: ARTN e67004 10.1371/journal.pone.0067004 PubMed PMID: ISI:000321738400093. 23826185 PMC3691263

[7] Smith ME, Singh BK, Michael E. Assessing endgame strategies for the elimination of lymphatic filariasis: A model-based evaluation of the impact of DEC-medicated salt. Scientific Reports. 2017;7. doi: Artn 7386 10.1038/S41598-017-07782-9 PubMed PMID: ISI:000407080100016. 28785097 PMC5547057

[8] Smith ME, Singh BK, Irvine MA, Stolk WA, Subramanian S, Hollingsworth TD, et al Predicting lymphatic filariasis transmission and elimination dynamics using a multi-model ensemble framework. Epidemics. 2017;18:16–28. 10.1016/j.epidem.2017.02.006 28279452 PMC5340857

[9] LaDeau SL, Glass GE, Hobbs NT, Latimer A, Ostfeld RS. Data–model fusion to better understand emerging pathogens and improve infectious disease forecasting. Ecological Applications. 2011;21(5):1443–60. 21830694 10.1890/09-1409.1 PMC7163730

[10] Luo Y, Ogle K, Tucker C, Fei S, Gao C, LaDeau S, et al Ecological forecasting and data assimilation in a data‐rich era. Ecological Applications. 2011;21(5):1429–42. 21830693 10.1890/09-1275.1

[11] Niu SL, Luo YQ, Dietze MC, Keenan TF, Shi Z, Li JW, et al The role of data assimilation in predictive ecology. Ecosphere. 2014;5(5). doi: Artn 65 10.1890/Es13-00273.1 PubMed PMID: ISI:000337164100016.

[12] Irvine MA, Stolk WA, Smith ME, Subramanian S, Singh BK, Weil GJ, et al Effectiveness of a triple-drug regimen for global elimination of lymphatic filariasis: a modelling study. The Lancet Infectious Diseases. 2017;17(4):451–8. 10.1016/S1473-3099(16)30467-4 28012943

[13] Britton T, Lindenstrand D. Epidemic modelling: Aspects where stochasticity matters. Mathematical Biosciences. 2009;222(2):109–16. 10.1016/j.mbs.2009.10.001 PubMed PMID: ISI:000273101900005. 19837097

[14] Weng E, Luo Y. Relative information contributions of model vs. data to short‐and long‐term forecasts of forest carbon dynamics. Ecological Applications. 2011;21(5):1490–505. 21830697 10.1890/09-1394.1

[15] Höhle M, Jørgensen E, O'Neill PD. Inference in disease transmission experiments by using stochastic epidemic models. Journal of the Royal Statistical Society: Series C (Applied Statistics). 2005;54(2):349–66.

[16] He D, Ionides EL, King AA. Plug-and-play inference for disease dynamics: measles in large and small populations as a case study. Journal of the Royal Society Interface. 2009. 10.1098/rsif.2009.0151 PMC2842609 19535416

[17] Beckage B, Gross LJ, Kauffman S. The limits to prediction in ecological systems. Ecosphere. 2011;2(11):1–12.

[18] Coveney PV, Dougherty ER, Highfield RR. Big data need big theory too. Phil Trans R Soc A. 2016;374(2080):20160153 10.1098/rsta.2016.0153 27698035 PMC5052735

[19] Clark JS, Gelfand AE. Hierarchical modelling for the environmental sciences: statistical methods and applications: Oxford University Press 2006.

[20] Ricciuto DM, King AW, Dragoni D, Post WM. Parameter and prediction uncertainty in an optimized terrestrial carbon cycle model: Effects of constraining variables and data record length. Journal of Geophysical Research: Biogeosciences. 2011;116(G1).

[21] Singh BK, Michael E. Bayesian calibration of simulation models for supporting management of the elimination of the macroparasitic disease, Lymphatic Filariasis. Parasites & Vectors. 2015;8:522. doi: Artn 522 10.1186/S13071-015-1132-7 PubMed PMID: ISI:000363306500001. 26490350 PMC4618871

[22] Chen M, Liu S, Tieszen L, Hollinger D. An improved state-parameter analysis of ecosystem models using data assimilation. ecological modelling. 2008;219(3–4):317–26.

[23] Arhonditsis GB, Papantou D, Zhang W, Perhar G, Massos E, Shi M. Bayesian calibration of mechanistic aquatic biogeochemical models and benefits for environmental management. Journal of Marine Systems. 2008;73(1–2):8–30.

[24] Keenan TF, Carbone MS, Reichstein M, Richardson AD. The model–data fusion pitfall: assuming certainty in an uncertain world. Oecologia. 2011;167(3):587 10.1007/s00442-011-2106-x 21901361

[25] Lipsitch M, Cohen T, Cooper B, Robins JM, Ma S, James L, et al Transmission dynamics and control of severe acute respiratory syndrome. Science. 2003;300(5627):1966–70. 10.1126/science.1086616 12766207 PMC2760158

[26] Lloyd-Smith JO, Galvani AP, Getz WM. Curtailing transmission of severe acute respiratory syndrome within a community and its hospital. Proceedings of the Royal Society of London B: Biological Sciences. 2003;270(1528):1979–89. 10.1098/rspb.2003.2481 PMC1691475 14561285

[27] Riley S, Fraser C, Donnelly CA, Ghani AC, Abu-Raddad LJ, Hedley AJ, et al Transmission dynamics of the etiological agent of SARS in Hong Kong: impact of public health interventions. Science. 2003;300(5627):1961–6. 10.1126/science.1086478 12766206

[28] Oreskes N, Belitz K. Philosophical issues in model assessment. Model validation: Perspectives in hydrological science. 2001;23.

[29] Dowd M. A sequential Monte Carlo approach for marine ecological prediction. Environmetrics. 2006;17(5):435–55.

[30] Kl Hsu, Moradkhani H, Sorooshian S. A sequential Bayesian approach for hydrologic model selection and prediction. Water Resources Research. 2009;45(12).

[31] Moniz L, Nichols J, Nichols JM, Cooch EG, Pecora L. Inferences About Coupling from Ecological Surveillance Monitoring: Approaches Based on Nonlinear Dynamics and Information Theory Towards an Information Theory of Complex Networks: Springer; 2011 p. 169–98.

[32] Keenan TF, Davidson E, Moffat AM, Munger W, Richardson AD. Using model‐data fusion to interpret past trends, and quantify uncertainties in future projections, of terrestrial ecosystem carbon cycling. Global Change Biology. 2012;18(8):2555–69.

[33] Keenan TF, Davidson EA, Munger JW, Richardson AD. Rate my data: quantifying the value of ecological data for the development of models of the terrestrial carbon cycle. Ecological Applications. 2013;23(1):273–86. PubMed PMID: ISI:000315104800021. 23495651 10.1890/12-0747.1

[34] Richardson AD, Williams M, Hollinger DY, Moore DJ, Dail DB, Davidson EA, et al Estimating parameters of a forest ecosystem C model with measurements of stocks and fluxes as joint constraints. Oecologia. 2010;164(1):25–40. 10.1007/s00442-010-1628-y 20390301

[35] Michael E, Bundy DAP. Global mapping of lymphatic filariasis. Parasitology Today. 1997;13(12):472–6. 10.1016/S0169-4758(97)01151-4 PubMed PMID: ISI:A1997YH63200008. 15275135

[36] Shea K, Tildesley MJ, Runge MC, Fonnesbeck CJ, Ferrari MJ. Adaptive management and the value of information: learning via intervention in epidemiology. PLoS biology. 2014;12(10):e1001970 10.1371/journal.pbio.1001970 25333371 PMC4204804

[37] Chadès I, Martin TG, Nicol S, Burgman MA, Possingham HP, Buckley YM. General rules for managing and surveying networks of pests, diseases, and endangered species. Proceedings of the National Academy of Sciences. 2011;108(20):8323–8. 10.1073/pnas.1016846108 PMC3100963 21536884

[38] Gambhir M, Bockarie M, Tisch D, Kazura J, Remais J, Spear R, et al Geographic and ecologic heterogeneity in elimination thresholds for the major vector-borne helminthic disease, lymphatic filariasis. Bmc Biol. 2010;8. doi: Artn 22 10.1186/1741-7007-8-22 PubMed PMID: ISI:000276298600001. 20236528 PMC2848205

[39] Irvine MA, Reimer LJ, Njenga SM, Gunawardena S, Kelly-Hope L, Bockarie M, et al Modelling strategies to break transmission of lymphatic filariasis—aggregation, adherence and vector competence greatly alter elimination. Parasites & Vectors. 2015;8. doi: Artn 547 10.1186/S13071-015-1152-3 PubMed PMID: ISI:000363307700001. 26489753 PMC4618540

[40] Jambulingam P, Subramanian S, de Vlas SJ, Vinubala C, Stolk WA. Mathematical modelling of lymphatic filariasis elimination programmes in India: required duration of mass drug administration and post-treatment level of infection indicators. Parasite Vector. 2016;9. doi: Artn 501 10.1186/S13071-016-1768-Y PubMed PMID: ISI:000383851100001. 27624157 PMC5022201

[41] Swaminathan S, Subash PP, Rengachari R, Kaliannagounder K, Pradeep DK. Mathematical models for lymphatic filariasis transmission and control: Challenges and prospects. Parasite Vector. 2008;1. doi: Artn 2 10.1186/1756-3305-1-2 PubMed PMID: ISI:000261100600001. 18275593 PMC2275317

[42] Plaisier AP, Subramanian S, Das PK, Souza W, Lapa T, Furtado AF, et al The LYMFASIM simulation program for modeling lymphatic filariasis and its control. Methods of Information in Medicine. 1998;37(1):97–108. PubMed PMID: ISI:000072593500014. 9550853

[43] Subramanian S, Stolk WA, Ramaiah KD, Plaisier AP, Krishnamoorthy K, Van Oortmarssen GJ, et al The dynamics of Wuchereria bancrofti infection: a model-based analysis of longitudinal data from Pondicherry, India. Parasitology. 2004;128:467–82. 10.1017/S0031182004004822 PubMed PMID: ISI:000221650700001. 15180315

[44] Organization WH. Monitoring and epidemiological assessment of mass drug administration in the global programme to eliminate lymphatic filariasis: a manual for national elimination programmes. 2011.

[45] Simonsen PE, Derua YA, Kisinza WN, Magesa SM, Malecela MN, Pedersen EM. Lymphatic filariasis control in Tanzania: effect of six rounds of mass drug administration with ivermectin and albendazole on infection and transmission. Bmc Infectious Diseases. 2013;13. doi: Artn 335 10.1186/1471-2334-13-335 PubMed PMID: ISI:000332223100001. 23870103 PMC3723586

[46] Ramaiah KD, Vanamail P, Pani SP, Yuvaraj J, Das PK. The effect of six rounds of single dose mass treatment with diethylcarbamazine or ivermectin on Wuchereria bancrofti infection and its implications for lymphatic filariasis elimination. Tropical Medicine & International Health. 2002;7(9):767–74. 10.1046/j.1365-3156.2002.00935.x PubMed PMID: ISI:000177950800009. 12225508

[47] Ramaiah KD, Vanamail P, Das PK. Changes in Wuchereria bancrofti infection in a highly endemic community following 10 rounds of mass administration of diethylcarbamazine. Transactions of the Royal Society of Tropical Medicine and Hygiene. 2007;101(3):250–5. 10.1016/j.trstmh.2006.05.007 PubMed PMID: ISI:000244130600007. 16890256

[48] Simonsen PE, Pedersen EM, Rwegoshora RT, Malecela MN, Derua YA, Magesa SM. Lymphatic Filariasis Control in Tanzania: Effect of Repeated Mass Drug Administration with Ivermectin and Albendazole on Infection and Transmission. Plos Neglected Tropical Diseases. 2010;4(6). doi: ARTN e696 10.1371/journal.pntd.0000696 PubMed PMID: ISI:000279341300005. 20532226 PMC2879369

[49] Stolk WA, De Vlas SJ, Borsboom GJJM, Habbema JDF. LYMFASIM, a simulation model for predicting the impact of lymphatic filariasis control: quantification for African villages. Parasitology. 2008;135(13):1583–98. 10.1017/S0031182008000437 PubMed PMID: ISI:000261548800010. 19006602

[50] Stolk WA, Swaminathan S, van Oortmarssen GJ, Das PK, Habbema JDF. Prospects for elimination of bancroftian filariasis by mass drug treatment in Pondicherry, India: A simulation study. Journal of Infectious Diseases. 2003;188(9):1371–81. 10.1086/378354 PubMed PMID: ISI:000186341400015. 14593597

[51] Spear RC, Hubbard A, Liang S, Seto E. Disease transmission models for public health decision making: Toward an approach for designing intervention strategies for Schistosomiasis japonica. Environmental Health Perspectives. 2002;110(9):907–15. PubMed PMID: ISI:000177893800033. 10.1289/ehp.02110907 12204826 PMC1240991

[52] Pardo L, Rovira-Esteva M, Busch S, Ruiz-Martin M, Tamarit JL, Unruh T. Bayesian Analysis of QENS data: From parameter determination to model selection. arXiv preprint arXiv:09073711. 2009.

[53] Peylin P, Bacour C, MacBean N, Leonard S, Rayner P, Kuppel S, et al A new stepwise carbon cycle data assimilation system using multiple data streams to constrain the simulated land surface carbon cycle. Geoscientific Model Development. 2016;9(9):3321.

[54] Fossum K, Mannseth T. Parameter sampling capabilities of sequential and simultaneous data assimilation: II. Statistical analysis of numerical results. Inverse Problems. 2014;30(11):114003.

[55] Stoy PC, Katul GG, Siqueira MBS, Juang JY, Novick KA, Uebelherr JM, et al An evaluation of models for partitioning eddy covariance-measured net ecosystem exchange into photosynthesis and respiration. Agricultural and Forest Meteorology. 2006;141(1):2–18. 10.1016/j.agrformet.2006.09.001 PubMed PMID: ISI:000243205000001.

[56] Kumar U, Kumar V, Kapur JN. Normalized Measures of Entropy. International Journal of General Systems. 1986;12(1):55–69. 10.1080/03081078608934927 PubMed PMID: ISI:A1986C691200004.

[57] Wang K, Phillips CA, Saxton AM, Langston MA. EntropyExplorer: an R package for computing and comparing differential Shannon entropy, differential coefficient of variation and differential expression. BMC research notes. 2015;8(1):832. 26714840 10.1186/s13104-015-1786-4 PMC4696313

[58] Ramaiah KD, Das PK, Vanamail P, Pani SP. Impact of 10 years of diethylcarbamazine and ivermectin mass administration on infection and transmission of lymphatic filariasis. Transactions of the Royal Society of Tropical Medicine and Hygiene. 2007;101(6):555–63. 10.1016/j.trstmh.2006.12.004 PubMed PMID: ISI:000246834400007. 17374389

[59] Benjamini Y, Hochberg Y. Controlling the False Discovery Rate—a Practical and Powerful Approach to Multiple Testing. J Roy Stat Soc B Met. 1995;57(1):289–300. PubMed PMID: ISI:A1995QE45300017.

[60] Richards FO, Eigege A, Miri ES, Kal A, Umaru J, Pam D, et al Epidemiological and entomological evaluations after six years or more of mass drug administration for lymphatic filariasis elimination in Nigeria. PLoS neglected tropical diseases. 2011;5(10):e1346 10.1371/journal.pntd.0001346 22022627 PMC3191131

[61] Madden LV, Hughes G, Bosch F. The study of plant disease epidemics: American Phytopathological Society (APS Press); 2007.

[62] Jewell CP, Brown RG. Bayesian data assimilation provides rapid decision support for vector-borne diseases. Journal of the Royal Society Interface. 2015;12(108):20150367. 10.1098/rsif.2015.0367 PMC4528604 26136225

[63] Walker M, Stolk WA, Dixon MA, Bottomley C, Diawara L, Traoré MO, et al Modelling the elimination of river blindness using long-term epidemiological and programmatic data from Mali and Senegal. Epidemics. 2017;18:4–15. 10.1016/j.epidem.2017.02.005 28279455 PMC5340858

[64] Michael E, Madon S. Socio-ecological dynamics and challenges to the governance of Neglected Tropical Disease control. Infectious diseases of poverty. 2017;6(1):35 10.1186/s40249-016-0235-5 28166826 PMC5292817

[65] Scheffer M, Bascompte J, Brock WA, Brovkin V, Carpenter SR, Dakos V, et al Early-warning signals for critical transitions. Nature. 2009;461(7260):53 10.1038/nature08227 19727193

[66] Drake JM, Griffen BD. Early warning signals of extinction in deteriorating environments. Nature. 2010;467(7314):456 10.1038/nature09389 20827269

[67] Erguler K, Stumpf MP. Practical limits for reverse engineering of dynamical systems: a statistical analysis of sensitivity and parameter inferability in systems biology models. Molecular BioSystems. 2011;7(5):1593–602. 10.1039/c0mb00107d 21380410

[68] Renzullo LJ, Barrett DJ, Marks AS, Hill MJ, Guerschman JP, Mu Q, et al Multi-sensor model-data fusion for estimation of hydrologic and energy flux parameters. Remote Sensing of Environment. 2008;112(4):1306–19.

