# Supplementary material for: Quantifying the value of surveillance data for improving model predictions of lymphatic filariasis elimination
Source: PLoS Negl Trop Dis. 2018 Oct 8;12(10):e0006674. doi: 10.1371/journal.pntd.0006674 (PMC6175292; doi:10.1371/journal.pntd.0006674)
Supplement: S1 Supplementary Information — (DOCX) [file pntd.0006674.s001.docx]

# S1 Supporting Information. Lymphatic filariasis model descriptions.

EPIFIL model description and methods

## The mathematical model of LF transmission dynamics

We employed a genus specific mosquito-vectored transmission model of LF to carry out the modelling work in this study ^1-7^. Briefly, the state variables of this hybrid coupled partial differential and differential equation model vary over age (*a*) and/or time (*t*), representing changes in the pre-patent worm burden per human host$(P(a,t)),$adult worm burden per human host$(W(a,t)),$the microfilariae (Mf) level in the human host modified to reflect infection detection in a 1 mL blood sample$(M(a,t)),$the average number of infective L3 larval stages per mosquito (*L*), and a measure of immunity$(I(a,t))$developed by human hosts against L3 larvae. The state equations comprising this model are:

The above equations involve partial derivatives of four state variables (*P* - pre-patent worm load; *W* - adult worm load; *M* - microfilaria intensity; *I* - immunity to acquiring new infection due to the pre-existing total worm load where *W_T_ = W(a,t) + P(a,t)*). Given the faster time scale of infection dynamics in the vector compared to the human host, the infective L3-stage larval density in mosquito population is modelled by an ordinary differential equation essentially reflecting the significantly faster time-scale of the infection dynamics in the vector hosts. This allows us to make the simplifying assumption that the density of infective stage larvae in the vector population reaches a dynamic equilibrium (denoted by *L^*^*) rapidly^1, 2, 5, 8, 9^. This basic coupled immigration-death structure of the model as well as its recent extensions has been extensively discussed previously^1-3, 5, 8, 9^. The effects of worm patency are captured by considering that at any time *t*, human individuals of age less than or equal to the pre-patency period, *τ*, will have no adult worms or Mf, and the rate at which pre-patent worms survive to become adult worms in these individuals at *a > τ* is given by $\zeta=\exp(-\mu\tau)$. The term enables us to account for the different establishment and development rates of the incoming L3-stage larvae as adult worms depending on the genus of mosquito vectors as expressed below:

$f[M(a,t)]=\left[\frac{2}{\left[1+\frac{M(a,t)}{k}\left(1-\exp\left[-\frac{r}{\kappa}\right]\right)\right]^{k}}-\frac{1}{\left[1+\frac{M(a,t)}{k}\left(1-\exp\left[-\frac{2r}{\kappa}\right]\right)\right]^{k}}\right]$ for mosquitoes of *Anopheline* genus;

$f[M(a,t)]=\left(1+\frac{M(a,t)}{k}\left(1-\exp\left[-\frac{r}{\kappa}\right]\right)\right)^{-k}$for mosquitoes of *Culicine* genus.

In the above, $k[=k_{0}+k_{Lin}M]$ is the shape parameter of the negative binomial distribution on the Mf uptake whereas r and are respectively the rate of initial increase and the maximum level of L3 larvae. See Table 1 for the description of all the model parameters and functions.

**Table 1** - **Description of EPIFIL model parameters and functions.**

| **Parameter** | **Definition (*units*)** | **Range** | **Refs** |
| --- | --- | --- | --- |
| ***λ*** | Number of bites per mosquito (*per month*) | [5, 15] | 1, 2, 5, 10, 11 |
| ***τ*** | Pre-patency period | [1, 9] | 12 |
| ***s*** | Proportion of female worms | 0.5 | - |
| ***μ*** | The worm mortality rate (*per month*) | [0.008, 0.018] | 1, 2, 5, 13-16 |
| ***α*** | Production rate of microfilariae per worm (*per month*) | [0.25, 1.5] | 1, 2, 5, 17 |
| ***γ*** | The death rate of the microfilariae (*per month*) | [0.08, 0.12] | 1, 5, 15, 17 |
| ***g*** | Proportion of mosquitoes which pick up infection when biting an infected host | [0.251, 0.485] | 1, 5, 18 |
| ***κ*** | Maximum level of L3 given Mf density | [3, 5] | 1, 5 |
| ***k_0_*** | The basic location parameter of negative binomial distribution used in aggregation parameter  ($k=k_{0}+k_{Lin}M$) | [0.000036, 0.000775] | 1, 5, 19, 20 |
| ***δ*** | Immunity waning rate (*per month*) | [0.001, 0.01] | 1, 5 |
| ***V/H*** | Ratio of number of vector to hosts | $MBR^{\#}/\lambda$ | data |
| ***k_Lin_*** | The linear rate of increase in the aggregation parameter defined above | [0.00000024, 0.282] | 1, 5, 19, 20 |
| ***σ*** | Death rate of mosquitoes (*per month*) | [1.5, 8.5] | 1, 5, 20 |
| ***ψ_1_*** | Proportion of L3 leaving mosquito per bite | [0.1, 0.8] | 17 |
| ***ψ_2_*** | The establishment rate^1^ | [0.00003, 0.00364] | 1, 2, 5, 21 |
| ***H_Lin_*** | A threshold value used in *h(a)* to adjust the rate at which individuals of age *a* are bitten: linear rise from 0 at age zero to 1 at age *H_Lin_* in years.$h(a)=a/H_{Lin}\text{ for }a<H_{Lin};h(a)=1\text{ for }a\geq H_{Lin}$ | [240, 360] months | 1, 5, 9 |
| ***r*** | Gradient of Mf uptake^2^ | [0.04, 0.25] | 1, 5 |
| ***c*** | Strength of acquired immunity | [0.015, 0.025] | 1, 5 |
| ***I_C_*** | Strength of immunosuppression^3^ | [0.5, 5.5] | 1, 5 |
| ***S_C_*** | Slope of immunosuppression function^4^  (*per worm/month*) | [0.01, 0.20] | 1, 5 |
| ***Intervention-related parameters*** | | | |
| ***ω*** | Worm killing efficacy of drug (instantaneous) | dependent on drug regimen | 3 |
| ***ε*** | Microfilariae killing efficacy of drug (instantaneous) | dependent on drug regimen | 3 |
| ***δ_reduc_*** | Reduction in the worm’s fecundity over a period of time *p* due to drug | dependent on drug regimen | 3 |
| ***p*** | A time period during which the drug remains efficacious in reducing the fecundity of the surviving adult worms | dependent on drug regimen | 3 |
| ***C*** | Percentage of the population administered the drug | data | data |
| ***MBR_VC_*** | Vector control (VC) modifies $V/H$($=MBR/\lambda$) where  $MBR_{VC}=MBR_{0}\exp[a_{1}t]$, with$a_{1}<0$for $\forall t$when VC is implemented, otherwise$a_{1}>0$. | data and estimates | 19, 20 |
| **Description** | **Mathematical expressions of the functions** | **Parameters** |  |
| Probability that an individual is of age *a* ***π(a)*** | $\pi(a)=A_{0}\exp[-B_{0}a]$ | Human age *a* in month, *A_0_* and *B_0_* estimated from country demographic data | 1, 5, 9 |
| Larvae establishment rate (modified by acquired immunity) ***Ω(a,t)*** | $L^{*}\psi_{1}\psi_{2}g_{1}(I)g_{2}(W_{T})$ | $\psi_{1}$- proportion of L3 leaving mosquito per bite; $\psi_{2}$- the establishment rate^1^ | - |
| Adult worm mating probability ***ϕ(W,k)*** | $1-\left(1+\frac{W}{2k}\right)^{-(1+k)}$ | *k* – negative binomial aggregation parameter | 2, 5, 22 |
| Immunity to larval establishment ***g_1_(I)*** | $\frac{1}{1+cI}$ | *c* – strength of immunity to larval establishment | 1, 5 |
| Host immunosuppression  ***g_2_(W_T_)*** | $\frac{1+I_{C}S_{C}W_{T}}{1+S_{C}W_{T}}$ | *I_C_* – strength of immunosuppression;  *S_C_* – slope of immunosuppression | 1, 5 |

^1^The proportion of L3-stage larvae infecting human hosts that survive to develop into adult worms^2^.

^2^The gradient of Mf uptake *r* is a measure of the initial increase in the infective L3 larvae uptake by vector as *M* increases from 0^2, 9^.

^3^ The facilitated establishment rate of adult worms due to parasite-induced immunosuppression in a heavily infected human host

^4^ The initial rate of increase by which the strength of immunosuppression is achieved as *W* increases from 0^23^.

# Note MBR (monthly biting rate) serves as an input to initialize the model, measured as mosquito bites per person per month, the value of which may be obtained from entomological surveys conducted in study sites. In the absence of the observed MBR value, the model has been adapted to estimate it from the community-level Mf prevalence data.

## Sequential fitting of the LF model to data

Typically, we follow a Bayesian Melding (BM) procedure of model fitting to data, outlined in detail in our previous work ^1, 2, 5, 6^. Under this framework, model fitting relies on the calculation of binomial log-likelihoods for quantifying how well a given parameter vector reproduces the observed infection prevalence in a population. A Sampling-Importance-Resampling (SIR) algorithm is then used to perform a specified number of draws with replacement to select the most likely parameter vectors describing the data from among a pool of parameter vectors. In this work, however, a chi-square goodness-of-fit metric was used across the groups for calibrating the models. We therefore replaced the binomial log-likelihood calculations and SIR algorithm with the calculation of a chi-square statistic and an acceptance/rejection step.

We began by using the known ranges of the parameter values to generate distributions of parameter priors. We then randomly sampled from these prior distributions to generate 100,000 parameter vectors, which were then used in the model with the observed ABR and intervention details (MDA times, coverages, and drug regimens) for a site to generate model outputs. The model outputs were melded with Mf prevalence data by calculating a chi-square statistic for each parameter vector where $\chi_{i}^{2}=\frac{(O_{ipos}-E_{ipos})^{2}}{\sigma_{i}^{2}}$ was the chi-square statistic for data point *i* where *O_i_* is the observed number of individuals testing positive for Mf at time *i*, *E_i_* is the model-predicted number of individuals testing positive for Mf at time *i* calculated as the model-predicted prevalence, *p*, multiplied by the number of individuals tested, *N*, and σ*_i_* is the experimental error associated with observed data *i* calculated as $\sqrt{p(1-p)N}$. All parameter vectors whose chi-square values are less than 3.841 (df=1, α=0.05) were accepted as satisfactorily reproducing the observed data and retained; all others were rejected. The accepted parameter vectors were used to generate distributions of variables of interest from the fitted model (eg. age-prevalence curves, worm breakpoints and infection trajectories following treatments). This fitting procedure was repeated sequentially for each observation considered in a particular data stream, effectively constraining the accepted parameter vectors with each new observation.

## Modeling intervention by mass drug administration

Intervention by mass drug administration was modeled based on the assumptions that anti-filarial treatment with a combination drug regimen acts by killing certain fractions of the populations of adult worms and microfilariae instantly after the drug administration. These effects are incorporated into the basic model by calculating the population sizes of worms and microfilariae as follows:

$$\left.\begin{matrix} P(a,t+dt)=(1-\omega C)P(a,t) \\ W(a,t+dt)=(1-\omega C)W(a,t) \\ M(a,t+dt)=(1-\varepsilon C)M(a,t) \end{matrix}\right\} \text{ at time }t=T_{MDA_{i}}$$

where *dt* is a short time period since the *i*th MDA was administered. During this short time interval, a given proportion of adult worms and microfilariae are instantly removed. The parameters *ω* and *ε* are drug killing efficacy rates for the two life stages of the parasite while the parameter *C* represents the MDA coverage. Apart from instantaneous killing of microfilariae, the drug continues to kill the newly reproduced Mf by any surviving adult worms at a rate *δ_reduc_* for a period of time, *p*. We model this effect as follows:

We simulated LF intervention by running the model with fixed values of *ε*, *δ_reduc_*, and *p* (here δ_reduc_ = 1) for MDA coverage levels given by data. The worm-kill parameter, *ω*, was drawn from a uniform prior distribution such that the post-intervention data could inform this efficacy value. The first MDA round was implemented in the model by affecting the population sizes of worms and microfilariae from the baseline fits, and then the intervention is simulated forward in time for a number of years, with subsequent MDA rounds implemented annually.

## References

1. Gambhir, M. and Michael, E. (2008) Complex ecological dynamics and eradicability of the vector borne macroparasitic disease, lymphatic filariasis. *PLoS One* **3**, e2874.

2. Gambhir, M. (2010) Geographic and ecologic heterogeneity in elimination thresholds for the major vector-borne helminthic disease, lymphatic filariasis. *BMC biology* **8**, 1.

3. Michael, E. *et al*. Mathematical modelling and the control of lymphatic filariasis. *The Lancet infectious diseases* **4**, 223-234 (2004).

4. Michael, E., Malecela-Lazaro, M. N., Kabali, C., Snow, L. C. & Kazura, J. W. Mathematical models and lymphatic filariasis control: endpoints and optimal interventions. *Trends Parasitol.* **22**, 226-233 (2006).

5. Singh BK and Bockarie MJ and Gambhir M and Siba PM and Tisch DJ and Kazura J and others. in *Sequential Modelling of the Effects of Mass Drug Treatments on Anopheline-Mediated Lymphatic Filariasis Infection in Papua New Guinea* (PLoS One, 2013).

6. Singh, B. K. & Michael, E. Bayesian calibration of simulation models for supporting management of the elimination of the macroparasitic disease, Lymphatic Filariasis. *Parasit vectors* **8**, 1-26 (2015).

7. Michael, E. & Singh, B. K. Heterogeneous dynamics, robustness/fragility trade-offs, and the eradication of the macroparasitic disease, lymphatic filariasis. *BMC medicine* **14**, 1 (2016).

8. Chan, M. S. *et al*. Epifil: a dynamic model of infection and disease in lymphatic filariasis. *Am. J. Trop. Med. Hyg.* **59**, 606-614 (1998).

9. Norman, R. *et al*. EPIFIL: the development of an age-structured model for describing the transmission dynamics and control of lymphatic filariasis. *Epidemiol. Infect.* **124**, 529-541 (2000).

10. Rajagopalan, P. Population dynamics of culex pipiens fatigans, the filariasis vector, in pondicherry: influence of climate and environment. *Proc Ind Nat Science Acad B* **46**, 745-752 (1980).

11. Subramanian, S., Manoharan, A., Ramaiah, K. D. & Das, P. K. Rates of acquisition and loss of Wuchereria bancrofti infection in Culex quinquefasciatus. *Am. J. Trop. Med. Hyg.* **51**, 244-249 (1994).

12. Scott, A. L. & Nutman, T. Lymphatic-dwelling filariae. *Lymphatic filariasis.*, 5-39 (2000).

13. Vanamail, P., Subramanian, S., Das, P. K., Pani, S. P. & Rajagopalan, P. K. Estimation of fecundic life span of Wuchereria bancrofti from longitudinal study of human infection in an endemic area of Pondicherry (south India). *Indian J. Med. Res.* **91**, 293-297 (1990).

14. Evans, D. B., Gelband, H. & Vlassoff, C. Social and economic factors and the control of lymphatic filariasis: a review. *Acta Trop.* **53**, 1-26 (1993).

15. Ottesen, E. & Ramachandran, C. Lymphatic filariasis infection and disease: control strategies. *Parasitology Today* **11**, 129-130 (1995).

16. Vanamail, P. *et al*. Estimation of the fecund life span of Wuchereria bancrofti in an endemic area. *Trans. R. Soc. Trop. Med. Hyg.* **90**, 119-121 (1996).

17. Hairston, N. G. & de Meillon, B. On the inefficiency of transmission of Wuchereria bancrofti from mosquito to human host. *Bull. World Health Organ.* **38**, 935-941 (1968).

18. Subramanian, S. *et al*. The relationship between microfilarial load in the human host and uptake and development of Wuchereria bancrofti microfilariae by Culex quinquefasciatus: a study under natural conditions. *Parasitology* **116**, 243-255 (1998).

19. Subramanian, S., Pani, S., Das, P. & Rajagopalan, P. Bancroftian filariasis in Pondicherry, south India: 2. Epidemiological evaluation of the effect of vector control. *Epidemiol. Infect.* **103**, 693-702 (1989).

20. Das, P. *et al*. Bancroftian filariasis in Pondicherry, south India–epidemiological impact of recovery of the vector population. *Epidemiol. Infect.* **108**, 483-493 (1992).

21. Ho, B. C. & Ewert, A. Experimental transmission of filarial larvae in relation to feeding behaviour of the mosquito vectors. *Trans. R. Soc. Trop. Med. Hyg.* **61**, 663-666 (1967).

22. May, R. M. Togetherness among schistosomes: its effects on the dynamics of the infection. *Math. Biosci.* **35**, 301-343 (1977).

23. Duerr, H., Dietz, K. & Eichner, M. Determinants of the eradicability of filarial infections: a conceptual approach. *Trends Parasitol.* **21**, 88-96 (2005).

LYMFASIM model description and methods

## Description of the mathematical model

LYMFASIM^1,2^ is a stochastic individual-based model for lymphatic filariasis (LF). It is a specific model variant within WORMSIM, a generalized framework for modelling transmission and control of helminth infections in humans^3,4^. LYMFASIM simulates the life histories of individual people and individual worms in a community, and the effects of interventions (e.g. mass drug administration, integrated vector management, bednet use) on transmission and morbidity, while taking into account the human demography and the complexities of helminth transmission. The model has been described elsewhere and has been applied to support decision making on control and elimination of lymphatic filariasis in different settings^1,2,5–13^.

Mass drug administration (MDA) is simulated by specifying the exact timing of the treatment rounds (year, month), the efficacy of the applied treatment regimen, the achieved coverage level, and compliance patterns. LYMFASIM assumes that a fraction of people never participates in MDA (e.g. systematic refusal, related to chronic illness). In addition, LYMFASIM allows the relative compliance to vary between age and sex groups; this mechanism captures transient contra-indications for MDA (e.g. exclusion of young children and pregnant women) and other age- and sex-related behavioral factors driving participation in MDA. Lastly, each individual has a personal inclination to participate in MDA, which is considered as a lifelong property. A stochastic process eventually defines for each individual whether they are treated in a given round, depending on the calculated probability.

## Parameter quantification and simulation methods for this study

The model was fitted to data from community intervention trials in Alagramam (India), Kirare (Tanzania) and Peneng (Papua New Guinea), as described in the main text.

To simulate Alagramam and Kirare, respectively, we used previously derived model parameterization for India^2^ and Africa^8^; the Africa quantification was also used to simulate LF in Papua New Guinea (PNG). The India and Africa/PNG model differ in the following aspects: age structure of the human population, density dependence in uptake by mosquito vectors (*Culex quinquefasciatus* in India; *Anopheles* species in Africa and PNG). Further, only the India model includes a form of acquired immunity (triggered by incoming L3 larvae and reducing the probability that incoming L3 larvae develop into adult worms). Treatment efficacy parameters vary between treatment regimens as listed in Table 3.

The strength of the anti-L3 immune response: in the original model, the strength of anti-L3 immunity parameter took the value of 0.0000589. This implies a strong immunity, which imposed a major restriction on the range of mf prevalence levels that could be simulated. Considering the relatively high mf prevalence in Alagramam and recognizing the uncertainty about the role of acquired immunity in LF transmission, for the purpose of this study we reduced the strength of immunity parameter value to 50% of its original value (see Table 2).

MDA timing and coverage (fraction of the population treated) was set to the reported coverage values for MDA rounds 1-6 and set to 65% for future rounds.

Parameter values are listed in Table 2 and Table 3. The following model parameters were estimated by fitting model predictions to data, as explained below

- Parameters describing local transmission conditions (i.e. monthly biting rate and exposure heterogeneity)
- The fraction of worms killed per treatment.

## Simulations

For each site, we performed a large number of simulations (30000 for Kirare, 20000 for Peneng, and 10000 for India) varying all free model parameters uniformly within the ranges specified in Tables 2 and 3. From this large set of simulations per site, we selected the runs that matched the criteria for each of the specified scenarios. For scenario 0, all runs with pre-control mf prevalence falling within prespecified ranges were selected. For scenario 1, a chi-square goodness of fit test was performed to compare the simulated and observed mf prevalence at baseline and all runs with a p-value above 0.05 were accepted. For scenarios 2-4 we applied chi-square goodness of fit test with Bonferroni correction to account for multiple testing when using more than one data point (accepting samples with p-value above 0.025 for scenarios 2/4, and above 0.0125 for scenario 3).

## Code availability

The code for the version of LYMFASIM used in this paper (version 2.58Ap26) is freely available on Gitlab [https://gitlab.com/erasmgz/wormsim.public/tags/2.58Ap26].

**Table 2. LYMFASIM input: probability distributions, functions and parameter values.**

| **Parameter description (symbol)** | **Model variant for India (Alagramam)** | | **Model variant for Africa (Kirare)** | | **Model variant for PNG (Peneng)** | | **Source / remarks** |
| --- | --- | --- | --- | --- | --- | --- | --- |
| **Human demography** |  |  |  |  |  |  |  |
| Cumulative survival (F(a)), by age | Age | Survival | Age | Survival | Age | Survival | Fixed, as in^2,8^ |
|  | 0 | 1 | 0 | 1 | 0 | 1 |
|  | 5 | 0.904 | 5 | 0.804 | 5 | 0.804 |
|  | 10 | 0.895 | 15 | 0.78 | 15 | 0.78 |
|  | 15 | 0.888 | 20 | 0.755 | 20 | 0.755 |
|  | 20 | 0.879 | 25 | 0.73 | 25 | 0.73 |
|  | 25 | 0.864 | 30 | 0.707 | 30 | 0.707 |
|  | 30 | 0.849 | 35 | 0.654 | 35 | 0.654 |
|  | 40 | 0.812 | 40 | 0.605 | 40 | 0.605 |
|  | 50 | 0.756 | 45 | 0.56 | 45 | 0.56 |
|  | 90 | 0 | 50 | 0.506 | 50 | 0.506 |
|  |  |  | 60 | 0.407 | 60 | 0.407 |
|  |  |  | 70 | 0.255 | 70 | 0.255 |
|  |  |  | 80 | 0.051 | 80 | 0.051 |
|  |  |  | 90 | 0 | 90 | 0 |
|  |  |  |  |  |  |  |  |
| Fertility rate per woman (R(a)), by age | Age | Fertility rate | Age | Fertility rate | Age | Fertility rate | Fixed, as in^2,8^ |
|  | 0 | 0 | 0 | 0 | 0 | 0 |
|  | 5 | 0 | 5 | 0 | 5 | 0 |
|  | 10 | 0 | 15 | 0 | 15 | 0 |
|  | 15 | 0 | 20 | 0.116 | 20 | 0.116 |
|  | 20 | 0.075 | 25 | 0.230 | 25 | 0.230 |
|  | 25 | 0.254 | 30 | 0.245 | 30 | 0.245 |  |
|  | 30 | 0.222 | 35 | 0.207 | 35 | 0.207 |  |
|  | 40 | 0.096 | 40 | 0.147 | 40 | 0.147 |  |
|  | 50 | 0.013 | 45 | 0.077 | 45 | 0.077 |  |
|  | 90 | 0 | 50 | 0.031 | 50 | 0.031 |  |
|  |  |  | 60 | 0 | 60 | 0 |  |
|  |  |  | 70 | 0 | 70 | 0 |  |
|  |  |  | 80 | 0 | 80 | 0 |  |
|  |  |  | 90 | 0 | 90 | 0 |  |
|  |  |  |  |  |  |  |  |
| Initial population | Age | Males/females | Age | Male/females | Age | Male/females | Assumed |
|  | 5 | 20/20 | 5 | 42/42 | 5 | 42/42 |  |
|  | 10 | 17/17 | 15 | 63/63 | 15 | 63/63 |  |
|  | 15 | 15/15 | 20 | 26/26 | 20 | 26/26 |  |
|  | 20 | 15/15 | 25 | 22/22 | 25 | 22/22 |  |
|  | 25 | 22/22 | 30 | 20/20 | 30 | 20/20 |  |
|  | 30 | 20/20 | 35 | 17/17 | 35 | 17/17 |  |
|  | 40 | 15/15 | 40 | 14/14 | 40 | 14/14 |  |
|  | 50 | 13/13 | 45 | 11/11 | 45 | 11/11 |  |
|  | 90 | 13/13 | 50 | 9/9 | 50 | 9/9 |  |
|  |  |  | 60 | 14/14 | 60 | 14/14 |  |
|  |  |  | 70 | 9/9 | 70 | 9/9 |  |
|  |  |  | 80 | 3/3 | 80 | 3/3 |  |
|  |  |  | 90 | 1/1 | 90 | 1/1 |  |
|  |  |  |  |  |  |  |  |
| Maximum population size | 3279 |  | 1000 |  | 1000 |  | Assumed |
| Proportion removed when maximum population size is reached | 5% |  | 5% |  | 5% |  | Assumed |
|  |  |  |  |  |  |  |  |
| **Exposure** |  |  |  |  |  |  |  |
| External force-of-infection at start of burn-in period | 2 |  | 2 |  | 2 |  | Assumed |
| Duration of external force-of-infection at start of burn-in period | 2 years |  | 2 years |  | 2 years |  | Assumed |

| Average mosquito biting rate for adult men (mbr)[[1]](#footnote-1) | varied uniformly over the range [500-2000] | | | varied uniformly over the range [100-2000] | | varied uniformly over the range [600-2000] | | |  |
| --- | --- | --- | --- | --- | --- | --- | --- | --- | --- |
| Seasonal variation | No |  | | No |  | No |  | |  |
| Variation in exposure by age (no difference assumed between sexes) | 0.26 at birth, linearly increasing to reach 1 at the age of 19.1 and constant at 1 from this age onwards | | | 0 at birth, linearly increasing to reach 1 at the age of 20 and constant at 1 from this age onwards | | 0 at birth, linearly increasing to reach 1 at the age of 20 and constant at 1 from this age onwards | | | Previously estimated by fitting to data^2^; adjusted for Africa/PNG^8^ |
| Probability distribution describing variation in the individual exposure index (Ei), due to personal factors (fixed through life) given age and sex | Gamma distribution with mean 1.0 and shape (=rate) varied uniformly with the range [0.15-5] | | | Gamma distribution with mean 1.0 and shape (=rate) varied uniformly with the range [0.15-4] | | Gamma distribution with mean 1.0 and shape (=rate) varied uniformly with the range [0.15-4] | | | Gamma distribution is assumed; shape/rate parameter to be estimated from data |
|  |  |  | |  |  |  |  | |  |
| **Parasite dynamics within host** |  |  | |  |  |  |  | |  |
| Success ratio (sr) | 0.00103 |  | | 0.00088 |  | 0.00088 | |  | Previously estimated by fitting to data^2,8^ |
| Anti-L3 immunity |  |  | |  |  |  |  | |  |
| - Shape-parameter for the gamma-distribution describing individual variation in the ability to develop an anti-L3 immune-response (rho-l) | 1.07 |  | | NA |  | NA |  | | Previously estimated by fitting to data^2,8^ |
| - Strength of immunological memory for anti-L3 immunity (gamma-l) | 0.00002945 | |  | NA |  | NA |  | | Assumed 50% lower than previously estimated^2^ |
| - Duration of immunological memory for anti-L3 immunity (THl), in years | 9.6 |  | | NA |  | NA |  | | Previously estimated by fitting to data^2^ |
| Anti-fecundity immunity: |  |  | |  |  |  |  | |  |
| - Shape-parameter for the gamma-distribution describing individual variation in the ability to develop an anti-fecundity immune-response (rho-w) | NA |  | | NA |  | NA |  | |  |
| - Strength of immunological memory for anti-fecundity immunity (gamma-w) | NA |  | | NA |  | NA |  | |  |
| - Duration of immunological memory for anti-fecundity immunity (THw), in years | NA |  | | NA |  | NA |  | |  |
|  |  |  | |  |  |  |  | |  |
| Average worm lifespan (Tl) | 10.2 on average; varied according to a Weibull distribution with shape 2 | | | 10 on average; varied according to a Weibull distribution with shape 2 | | 10 on average; varied according to a Weibull distribution with shape 2 | | | Previously estimated by fitting to data^2^ |
| Duration of immature stage of the parasite in human host (Ti) | Constant, 8 months | | | Constant, 8 months | | Constant, 8 months | | | Fixed^14^ |
| No. of Mf produced per female parasite per month per 20 ml peripheral blood in the absence of immune reactions and in the presence of at least 1 male worm (r0) | 0.606 |  | | 0.58 |  | 0.58 |  | | Previously estimated by fitting to data^2^ |
| Monthly survival of the microfilariae, fraction (s) | 0.9 |  | | 0.9 |  | 0.9 |  | | Fixed, based on^15^ |
| Association between worm age and mf production rate | mf production independent of worm age | | | mf production independent of worm age | | mf production independent of worm age | | | Assumed |
| Polygamy (all female worms produce mf in the presence of at least one male worm) | Yes (male potential 1000) | | | Yes (male potential 1000) | | Yes (male potential 1000) | | |  |
| Mating cycle (number of months a female can produce mf with one insemination) | 1 |  | | 1 | | 1 | | | Assumed |
| **Uptake of infection by the vector** |  |  | |  | |  | | |  |
| Functional relationship[[2]](#footnote-2) | $L3=c+\frac{aM}{1+aM/(b-c)}$ | | | $L3=a(1-\exp\left( -(bM \right)^{c}))$ | | $L3=a(1-\exp\left( -(bM \right)^{c}))$ | | | Previously estimated by fitting model to data ^2,8,16^ |
|  | a | 0.089 | | a | 1.666 | a | 1.666 | |
|  | b | 6.6 | | b | 0.027 | b | 0.027 | |
|  | c | 0 | | c | 1.514 | c | 1.514 | |
| Transmission probability (v), fraction of the L3 larvae, resulting from a single blood meal, that is released by a mosquito | 0.1 |  | | 0.1 |  | 0.1 |  | | Fixed, as in^2^ |
| **Other** |  |  | |  |  |  |  | |  |
| Duration of warming up period | 144 |  | | 150 |  | 150 |  | | Assumed |
|  |  |  | |  |  |  |  | |  |
| **Surveillance** |  |  | |  |  |  |  | |  |
| Timing of surveys | Monthly from 1^st^ treatment onwards | | | Monthly from 1^st^ treatment onwards | | Monthly from 1^st^ treatment onwards | | |  |
| Volume of blood examined for mf | 60 μL |  | | 100 μL | | 1 mL^c^ |  | | Data |
| Variability in observed number of mf in one 20 μl blood smear | Negative binomial distribution with k=0.345 | | | Negative binomial distribution with k=0.033 | | Negative binomial distribution with k=0.033 | | | Previously estimated for 20 μL blood by fitting to data^2^, assumed for Africa and PNG |
| Variation between worms in their contribution to measured mf count (dispersal factor) | Constant (no variation) | | | Constant (no variation) | | Constant (no variation) | | | Assumed |
|  |  |  | |  |  |  |  | |  |
| **Morbidity** |  |  | |  |  |  |  | |  |
| not applicable |  |  | |  |  |  |  | |  |
| no excess mortality due to disease |  |  | |  |  |  |  | |  |

**Table 3. LYMFASIM assumptions related to MDA and treatment efficacy**

| **Parameter description (symbol)** | **Model variant for India (Alagramam)** | **Model variant for Africa (Kirare)** | **Model variant for PNG (Peneng)** |
| --- | --- | --- | --- |
| Timing and coverage of treatments | As reported | As reported | As reported |
| Compliance |  |  |  |
| Fraction excluded from treatment | 0 | 0 | 0 |
| Minimum age for treatment | 2 years | 5 years | 5 years |

| Relative compliance by age and sex | Default pattern, as in[[3]](#footnote-3) | Default pattern | Default pattern |
| --- | --- | --- | --- |
| Treatment effects |  |  |  |
| Treatment regimen | DEC | IVER+ALB | DEC+IVER |
| Fraction malabsorption | 0 | 0 | 0 |
| Permanent reduction mf-production | 0 | 0 | 0 |
| Period of recovery (years) | 0.25 | 0.75 | 0.75 |
| Shape parameter recovery function^a^ | 1 | 1 | 1 |
| Fraction killed | varied over the range [0.1-0.85] | varied over the range [0.1-0.85] | varied over the range [0.1-0.85] |
| Treatment effect variability | NA (assumed constant, 1) | NA (assumed constant, 1) | NA (assumed constant, 1) |
| Fraction mf surviving | Constant 0.1 | Constant, 0.01 | Constant, 0.01 |

The default pattern is as follows:

| **Age** | **Females** | **Males** |
| --- | --- | --- |
| Minimum age for treatment (2 or 5 depending on the treatment regimen) | 0 | 0 |
| 10 | 0.75 | 0.75 |
| 15 | 0.80 | 0.70 |
| 20 | 0.80 | 0.74 |
| 30 | 0.70 | 0.65 |
| 50 | 0.75 | 0.70 |
| 60 | 0.75 | 0.70 |
| 99 | 0.80 | 0.75 |

## References

1 Plaisier AP, Subramanian S, Das PK (1998)*.* The LYMFASIM simulation program for modeling lymphatic filariasis and its control. Methods Inf Med 37: 97–108.

2 Subramanian S, Stolk WA, Ramaiah KD (2004)The dynamics of *Wuchereria bancrofti* infection: a model-based analysis of longitudinal data from Pondicherry, India. Parasitolog*y* 128: 467–482.

3 Coffeng LE, Bakker R, Montresor A, de Vlas SJ (2015) Feasibility of controlling hookworm infection through preventive chemotherapy: a simulation study using the individual-based WORMSIM modelling framework. Parasit Vectors 8: 541.

4 Stolk W, Walker M, Coffeng L, Basáñez M-G, Vlas S. Required duration of mass ivermectin treatment for onchocerciasis elimination in Africa: a comparative modelling analysis. *Parasit Vectors* 2015; **8**: 552.

5 Stolk WA, Swaminathan S, Oortmarssen GJ van, Das PK, Habbema JDF (2003) Prospects for elimination of bancroftian filariasis by mass drug treatment in Pondicherry, India: a simulation study. J Infect Dis 188: 1371–1381.

6 Stolk WA, de Vlas SJ, Habbema JDF (2005) Anti-Wolbachia treatment for lymphatic filariasis. Lancet Infect Dis 365: 2067–2068.

7 Stolk WA, De Vlas SJ, Habbema JDF. Advances and challenges in predicting the impact of lymphatic filariasis elimination programmes by mathematical modelling. *Filaria J* 2006; **5**: 5.

8 Stolk WA, De Vlas SJ, Borsboom GJ, Habbema JDF (2008) LYMFASIM, a simulation model for predicting the impact of lymphatic filariasis control: quantification for African villages. Parasitology 135: 1583–1598.

9 Stolk WA, ten Bosch QA, de Vlas SJ, Fischer PU, Weil GJ (2013) Modeling the impact and costs of semiannual mass drug administration for accelerated elimination of lymphatic filariasis. PLoS Negl Trop Dis **7**: e1984.

10 Stolk WA, Stone C, de Vlas SJ (2015) Modelling lymphatic filariasis transmission and control: modelling frameworks, lessons learned and future directions. Adv Parasito*l* 87: 249–291.

11 Jambulingam P, Subramanian S, de Vlas SJ, Vinubala C, Stolk WA (2016) Mathematical modelling of lymphatic filariasis elimination programmes in India: required duration of mass drug administration and post-treatment level of infection indicators. Parasit Vectors 9: 501.

12 Irvine MA, Stolk WA, Smith ME (2017) Effectiveness of a triple-drug regimen for global elimination of lymphatic filariasis: a modelling study. Lancet Infect Dis 17: 451–458.

13 Smith ME, Singh BK, Irvine MA (2017) Predicting lymphatic filariasis transmission and elimination dynamics using a multi-model ensemble framework. Epidemics 18: 16–28.

14 World Health Organization (1992) Lymphatic filariasis: the disease and its control. Fifth report of the WHO Expert Committee on Filariasis. World Health Organ Tech Rep Se*r.* 821: 1–71.

15 Plaisier AP, Cao WC, Van Oortmarssen GJ, Habbema JDF. Efficacy of ivermectin in the treatment of *Wuchereria bancrofti* infection: a model-based analysis of trial results. Parasitology 119: 385–394.

16 Subramanian S, Krishnamoorthy K, Ramaiah KD, Habbema JDF, Das PK, Plaisier AP (1998) The relationship between microfilarial load in the human host and uptake and development of *Wuchereria bancrofti* microfilariae by *Culex quinquefasciatus*: a study under natural conditions. Parasitology 116: 243–255.

TRANSFIL model description and methods

## Description of the mathematical model

The mathematical model of lymphatic filariasis (LF) transmission TRANSFIL is a stochastic individual-based model of LF infection in human populations. A full model description is given in Irvine *et al.*^1^ and more recently in Smith *et al.*^2^, so here we provide a brief summary of the model development. The model is a stochastic micro-simulation of individuals with worm burden, microfilaraemia and other demographic parameters relating to age and risk of exposure. Humans are modelled individually, with their own male and female worm burden. The concentration of mf in the peripheral blood is modelled for each individual and increases according to the number of fertile female worms as well as decreasing at constant rate.

The total mf density in the population contributes towards the current density of L3 larvae in the human-biting mosquito population, where the distribution of L3 amongst the human- biting mosquito population is completely homogeneous. An empirically derived relationship is used for the uptake of mf by a mosquito, where both *Culex* and *Anopheles* uptake curves are implemented depending on setting. The model dynamics are therefore divided into the individual human dynamics, including age and turnover; worm dynamics inside the host; microfilariae dynamics inside the host and larvae dynamics inside the mosquito.

Finally, the model includes a very low importation rate in order to ensure that the equilibrium distribution exists. The interventions reduce the prevalence over time, and therefore as year pass, the importation rate decreases based on some pilot simulations. See Table 4 for the description of the model parameters.

**Table 4: Description the basic LF model parameters.**

| **Parameter symbol** | **Definition** | **Value** | **Source** |
| --- | --- | --- | --- |
| λ | Number of bites per mosquito | 10 per month | 3,4 |
| V/H | Ratio of number of vectors to hosts | Varied | Data |
| α_max_ | Age at which exposure to mosquitoes reaches its maximum level | 20.0 | 5 |
| ψ_1_ | Proportion of L3 leaving mosquito per bite | 0.414 | 6 |
| ψ_2_ | Proportion of L3 leaving mosquito that enter host | 0.32 | 7 |
| s_2_ | Proportion of L3 entering host that develop into adult worms | 0.00275 | 8,9 |
| μ | Death rate of adult worms | 0.0104 per month | 10 |
| α | Production rate of Mf per worm | 0.2 per month | 6 |
| γ | Death rate of Mf | 0.1 per month | 6,11 |
| g | Proportion of mosquitoes which pick up infection when biting an infected host | 0.37 | 12 |
| σ | Death rate of mosquitoes | 5 per month | 7 |
| k | Aggregation parameter of individual exposure to mosquitoes | Varied | Data |
| h(α) | Parameter to adjust rate at which individuals of age α are bitten | Linear from 0 to 10, with maximum of 1 | 8 |
| a | Importation rate per month | Varied uniformly over the range [0, 0.0005] | N/A |

## Modelling intervention by Mass Drug Administration (MDA)

The effect of MDA is simulated for an individual by reducing their mf concentration and their male and female worm burden according to the efficacy of the treatment. In addition, there is a period after MDA during which the production of mf for that individual is diminished. Furthermore, we modelled individuals' compliance after multiple rounds of treatment based on the paper by Griffin *et al.*^13^, where a parameter ρ is used to model the probability of an individual making the same decision than in the previous round of treatment (see Irvine *et al.*^1^ for more details). This approach is different from the other models in this manuscript, and they have been compared recently by Dyson *et al.*^14^. We selected the ρ value for our simulations by using maximum likelihood to minimize the difference in the distribution of the number of rounds attended after ten treatments when compared to the semi-systematic approach taken by Stolk *et al.*^15^ and described in Dyson *et al.*^14^.

## Simulation methods for this study

Model fitting was performed on data collected in three districts: Alagramam (India), Kirare (Tanzania) and Peneng (Papua New Guinea, PNG). The main differences among these three regions are the dominant vector species, *Culex* for India and *Anopheles* for Tanzania and PNG, the relative ranges of prevalence that we considered (based on the information available), and the treatment regimens. The main three aspects that differentiate the treatments are the mortality caused in adult’s worms and microfilariae, and the sterilization of the surviving adults. These are summarized in the main manuscript.

To generate the require range of MF prevalences for each site, we varied three parameters of the model, the vector to host ratio (V/H), the average population bite risk (k) and the importation rate (a), using parameter sets from a range of plausible values based on previously analysed data for each of the three districts. More specifically, we varied these model parameters uniformly within the ranges shown in Table 5. For each region, 100.000 parameter values were drawn from these prior distributions, which are then used to generate simulations from the model using the exact times and coverage of the MDA treatments. For each of the specified scenarios listed in the main text, we selected the simulations that matched the mf prevalence seen in the observed data using the chi-squared test statistic with a significance level of 0.05.

**Table 5: Possible values of V/H, k and a for which the required mf prevalence levels are achieved for each of the three sites.**

| **Parameter symbol** | **India** | **Africa** | **PNG** |
| --- | --- | --- | --- |
| k | Varied uniformly over the range [0.01, 0.20] | Varied uniformly over the range [0.01, 0.40] | Varied uniformly over the range [0.20, 0.80] |
| V/H | Varied uniformly over the range [0, 150] | Varied uniformly over the range [13, 22] | Varied uniformly over the range [50, 85] |
| a | Varied uniformly over the range [0, 0.0005] | Varied uniformly over the range [0, 0.0005] | Varied uniformly over the range [0, 0.0005] |

## References

1. Irvine MA, Reimer LJ, Njenga SM, Gunawardena S, Kelly-Hope L, Bockarie M, and Hollingsworth TD (2015) Modelling strategies to break transmission of lymphatic filariasis - aggregation, adherence and vector competence greatly alter elimination. Parasites and Vectors 8:547.

2. Smith ME, Singh BK, Irvine MA, Stolk WA, Subramanian S, Hollingsworth TD, and Michael E (2017) Predicting lymphatic filariasis transmission and elimination dynamics using a multi-model ensemble framework. Epidemics 18: 16-28.

3. Rajagopalan P (1980) Population dynamics of culex pipiens fatigans, the filariasis vector, in pondicherry: influence of climate and environment. Proc Indian Natl Sci Acad. 6**:** 745–752.

4. Subramanian S, Manoharan A, Ramaiah K, Das P (1994) Rates of acquisition and loss of wuchereria bancrofti infection in culex quinquefasciatus. Am J Trop Med Hyg. 51: 244–249.

5. Subramanian S, Stolk W, Ramaiah K, Plaisier A, Krishnamoorthy K, Van Oortmarssen G (2004). The dynamics of wuchereria bancrofti infection: a model-based analysis of longitudinal data from Pondicherry, India. Parasitology 128: 467–482.

6. Hairston NG, de Meillon B (1968) On the inefficiency of transmission of wuchereria bancrofti from mosquito to human host. Bull World Health Organ. 38: 935.

7. Ho BC, Ewert A (1967) Experimental transmission of filarial larvae in relation to feeding behaviour of the mosquito vectors. Trans R Soc Trop Med Hyg. 61: 663–666.

8. Norman R, Chan MS, Srividya A, Pani S, Ramaiah KD, Vanamail P (2000) EPIFIL: The development of an age-structured model for describing the transmission dynamics and control of lymphatic filariasis. Epidemiol Infect. 124: 529–541.

9. Stolk WA, De Vlas SJ, Borsboom GJ, Habbema J (2008) LYMFASIM, a simulation model for predicting the impact of lymphatic filariasis control: Quantification for African villages. Parasitology 135L: 1583–1598.

10. Evans DB, Gelband H, Vlassoff C (1993) Social and economic factors and the control of lymphatic filariasis: a review. Acta Trop. 53: 1–26.

11. Ottesen E, Ramachandran C (1995) Lymphatic filariasis infection and disease: control strategies. Parasitol Today 11: 129–130.

12. Subramanian S, Krishnamoorthy K, Ramaiah K, Habbema J, Das P, Plaisier A (1998) The relationship between microfilarial load in the human host and uptake and development of wuchereria bancrofti microfilariae by culex quinquefasciatus: a study under natural conditions. Parasitology. 116: 243–255.

13. Griffin JT, Hollingsworth TD, Okell LC, Churcher TS, White M, Hinsley W, Bousema T, Drakeley CJ, Ferguson NM, Basez MG, and Ghani AC. (2010) Reducing Plasmodium falciparum malaria transmission in Africa: a model-based evaluation of intervention strategies. PLOS Medicine. **7**: 1-17.

14. Dyson L, Stolk WA, Farrell SH, and Hollingsworth TD (2017) Measuring and modelling the effects of systematic non-adherence to mass drug administration. Epidemics 18: 56-66.

15. Stolk WA, Swaminathan S, van Oortmarssen GJ, Das PK, and Habbema JDF (2003) Prospects for elimination of bancroftian filariasis by mass drug treatment in Pondicherry, India: a simulation study. The Journal of Infectious Diseases 188: 1371-1381.

1. The relative biting rate (rbr) is fixed at 1 in all models [↑](#footnote-ref-1)
2. The uptake function for India can also be parameterized as follows: L3= $\frac{\varphi M}{1+\xi M}$, with ξ = 0.013485 and $\varphi$= 0.089

   ^c^ LYMFASIM has been parametrized for mf counts in 20 µl bloodsmears. However, it has been shown that 50 20-µl bloodsmears would overestimate mf counts in 1 mL of venous blood and therefore we assumed 20 bloodmsears of 20-µl. For literature on the relation between mf counts in capillary and venous blood see, e.g., Eberhard, M. L., et al. "Comparative densities of Wuchereria bancrofti microfilaria in paired samples of capillary and venous blood." Tropical medicine and parasitology: official organ of Deutsche Tropenmedizinische Gesellschaft and of Deutsche Gesellschaft fur Technische Zusammenarbeit (GTZ) 39.4 (1988): 295-298. [↑](#footnote-ref-2)
3. ^a^ where s is the shape parameter of the recovery function, indicates the reproductive capacity of female worm $j$ in month $t$, $\tau$ months after the last treatment and is the reproductive capacity of female worm $j$ had person $i$ not been treated at the last round $\tau$ months ago. The drug will reduce the reproductive capacity of female worm by a proportion $d_{i}$ in host $i$. The reproductive capacity will restore within a period $Tr_{i}$ to its maximum value. [↑](#footnote-ref-3)
