# Supplementary material for: Quantifying the value of surveillance data for improving model predictions of lymphatic filariasis elimination
Source: PLoS Negl Trop Dis. 2018 Oct 8;12(10):e0006674. doi: 10.1371/journal.pntd.0006674 (PMC6175292; doi:10.1371/journal.pntd.0006674)
Supplement: S2 Supplementary Information — (DOCX) [file pntd.0006674.s002.docx]

# S2 Supporting Information. Change in the distributions of model-predicted timelines to achieve elimination.


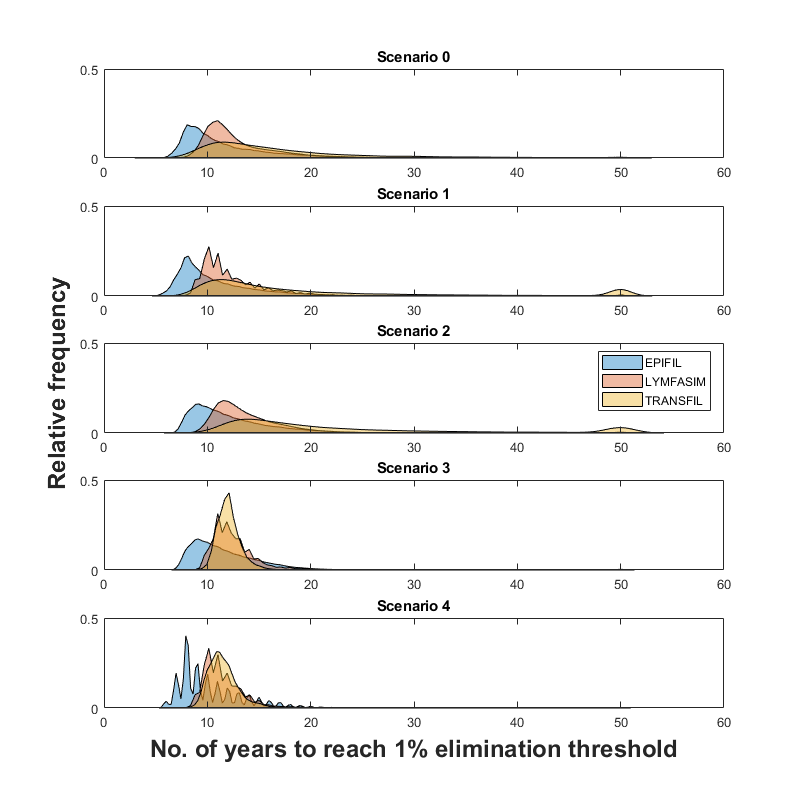

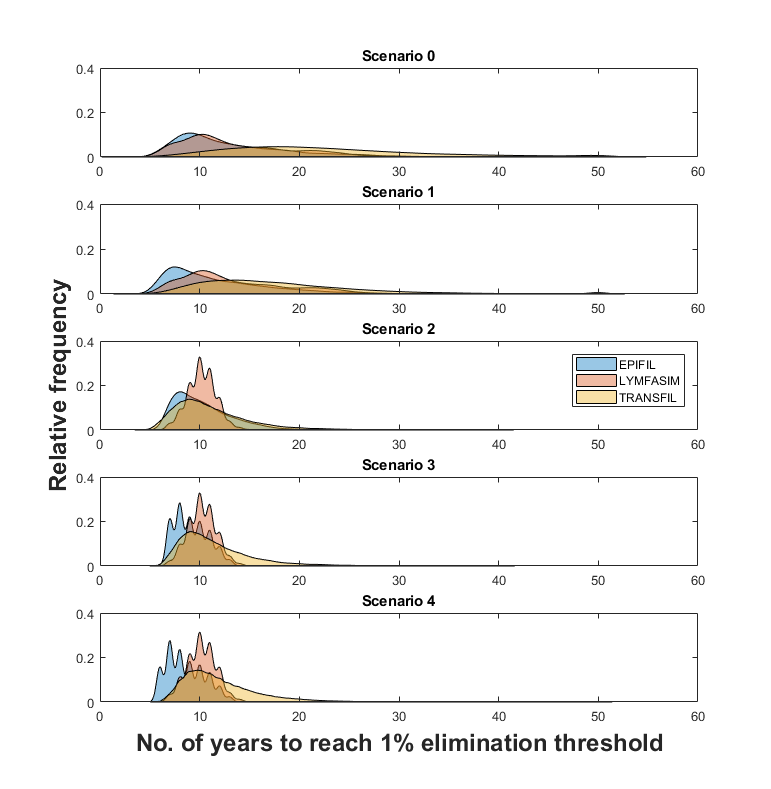


B

A

**Fig 1. Comparison of the distributions of predicted timelines to parasite elimination from the three LF models for Alagramam, India (A) and Peneng, PNG (B).** The results for both sites show that the predictions coming from the model-only simulations (Scenario 0) have the widest spread in their distributions across all three models, with addition of sequential data systematically reducing the uncertainty in the corresponding model predictions. Pairwise Kolmogorov-Smirnov tests for equal distributions were performed on the results from each model to evaluate whether updating the models with data changed the distribution of predictions (see Table 1 in S2 Supplementary Information).

## Table 1. Pairwise Kolmogorov-Smirnov tests for equal distributions.

| Scenario A | Scenario B | Kolmogorov-Smirnov *p*-values | | | | | | | | |
| --- | --- | --- | --- | --- | --- | --- | --- | --- | --- | --- |
|  |  | Kirare | | | Alagramam | | | Peneng | | |
|  |  | EPIFIL | LYMFASIM | TRANSFIL | EPIFIL | LYMFASIM | TRANSFIL | EPIFIL | LYMFASIM | TRANSFIL |
| 0 | 1 | **< 0.0001** | **< 0.0001** | **< 0.0001** | **< 0.0001** | **< 0.0001** | **< 0.0001** | **< 0.0001** | 0.999 | **< 0.0001** |
| 0 | 2 | **< 0.0001** | **< 0.0001** | **< 0.0001** | **< 0.0001** | **< 0.0001** | **< 0.0001** | **< 0.0001** | **< 0.0001** | **< 0.0001** |
| 0 | 3 | **< 0.0001** | **< 0.0001** | **< 0.0001** | **< 0.0001** | **< 0.0001** | **< 0.0001** | **< 0.0001** | **< 0.0001** | **< 0.0001** |
| 0 | 4 | **< 0.0001** | **< 0.0001** | **< 0.0001** | **< 0.0001** | **< 0.0001** | **< 0.0001** | **< 0.0001** | **< 0.0001** | **< 0.0001** |
| 1 | 2 | **< 0.0001** | **< 0.0001** | **< 0.0001** | **< 0.0001** | **< 0.0001** | **< 0.0001** | **< 0.0001** | **< 0.0001** | **< 0.0001** |
| 1 | 3 | **< 0.0001** | **< 0.0001** | **< 0.0001** | **< 0.0001** | **< 0.0001** | **< 0.0001** | **< 0.0001** | **< 0.0001** | **< 0.0001** |
| 1 | 4 | **< 0.0001** | **< 0.0001** | **< 0.0001** | **< 0.0001** | **< 0.0001** | **< 0.0001** | **< 0.0001** | **< 0.0001** | **< 0.0001** |
| 2 | 3 | 0.790 | **< 0.0001** | **< 0.0001** | **< 0.0001** | **< 0.0001** | **< 0.0001** | **< 0.0001** | 1.000 | **< 0.0001** |
| 2 | 4 | **< 0.0001** | **< 0.0001** | **< 0.0001** | **< 0.0001** | **< 0.0001** | **< 0.0001** | **< 0.0001** | 0.840 | **< 0.0001** |
| 3 | 4 | **0.002** | 0.203 | **< 0.0001** | **< 0.0001** | **< 0.0001** | **< 0.0001** | **< 0.0001** | 0.585 | **< 0.0001** |

Note: Bolded *p*-values are significant according to the Benjamini-Hochberg procedure for controlling the false discovery rate (*q* = 0.05).
