# Supplementary material for: Quantifying the value of surveillance data for improving model predictions of lymphatic filariasis elimination
Source: PLoS Negl Trop Dis. 2018 Oct 8;12(10):e0006674. doi: 10.1371/journal.pntd.0006674 (PMC6175292; doi:10.1371/journal.pntd.0006674)
Supplement: S3 Supplementary Information — (DOCX) [file pntd.0006674.s003.docx]

# **S3 Supporting Information. Spearman parameter correlations for Kirare and Peneng.**

### Table 1. Spearman parameter correlations for scenarios 1 (lower left triangle) and 3 (upper right triangle) for Kirare, Tanzania.

|  | λ | α | k_0_ | k_Lin_ | κ | r | σ | Ψ_1_ | Ψ_2_ | μ | γ | g | c | H_Lin_ | I_c_ | S_c_ | τ | δ |
| --- | --- | --- | --- | --- | --- | --- | --- | --- | --- | --- | --- | --- | --- | --- | --- | --- | --- | --- |
| λ |  | **-0.242** | 0.078 | -0.125 | 0.065 | -0.021 | 0.078 | -0.055 | -0.079 | -0.042 | -0.020 | 0.033 | -0.099 | -0.022 | -0.072 | 0.065 | 0.056 | 0.116 |
| α | -0.063 |  | 0.031 | **0.195** | **-0.203** | -0.123 | -0.048 | -0.015 | -0.186 | **0.258** | 0.167 | -0.185 | **0.313** | 0.108 | 0.059 | -0.070 | -0.003 | -0.134 |
| k_0_ | -0.028 | -0.012 |  | -0.075 | 0.042 | 0.052 | 0.059 | -0.068 | **-0.252** | -0.083 | -0.116 | 0.089 | 0.030 | -0.093 | 0.093 | 0.055 | 0.012 | -0.001 |
| k_Lin_ | -0.009 | 0.018 | -0.052 |  | **-0.212** | **0.242** | 0.039 | 0.067 | 0.004 | **0.292** | 0.151 | -0.020 | 0.064 | 0.008 | -0.164 | -0.086 | -0.160 | **-0.197** |
| κ | **0.128** | 0.059 | 0.034 | 0.021 |  | 0.027 | -0.035 | -0.002 | 0.182 | 0.043 | 0.010 | 0.002 | -0.123 | -0.043 | -0.032 | 0.148 | -0.133 | 0.067 |
| r | -0.068 | **-0.084** | 0.045 | 0.013 | **0.103** |  | 0.153 | 0.002 | -0.018 | 0.187 | 0.151 | -0.003 | -0.071 | 0.063 | 0.021 | 0.013 | -0.172 | -0.031 |
| σ | **-0.098** | **-0.178** | -0.023 | 0.022 | 0.061 | -0.039 |  | **0.195** | 0.002 | -0.035 | -0.104 | 0.145 | **-0.211** | 0.116 | 0.100 | -0.032 | -0.003 | **-0.201** |
| Ψ_1_ | 0.022 | **0.176** | 0.054 | **0.068** | -0.032 | **0.069** | **0.081** |  | 0.031 | 0.191 | **0.241** | 0.011 | 0.068 | -0.155 | -0.043 | -0.043 | -0.027 | -0.019 |
| Ψ_2_ | 0.050 | **0.201** | -0.011 | **0.081** | **-0.094** | **0.093** | **0.151** | **-0.168** |  | 0.154 | 0.120 | -0.172 | 0.108 | 0.061 | **0.215** | -0.105 | -0.178 | -0.081 |
| μ | -0.040 | **-0.081** | 0.030 | 0.060 | **0.072** | -0.048 | -0.043 | **0.189** | **0.187** |  | 0.098 | 0.170 | 0.104 | 0.067 | 0.063 | -0.005 | -0.136 | 0.143 |
| γ | -0.016 | **0.131** | -0.019 | **0.156** | -0.043 | 0.002 | 0.007 | -0.023 | -0.033 | -0.002 |  | 0.160 | 0.070 | **-0.368** | -0.018 | -0.096 | 0.009 | 0.001 |
| g | -0.015 | **0.146** | 0.020 | 0.049 | -0.009 | <0.001 | 0.047 | -0.035 | **-0.134** | 0.060 | -0.008 |  | -0.023 | 0.067 | 0.011 | 0.077 | 0.179 | -0.081 |
| c | -0.010 | -0.031 | 0.047 | **-0.219** | 0.015 | 0.024 | 0.019 | 0.011 | 0.029 | 0.023 | -0.047 | -0.045 |  | -0.095 | 0.177 | -0.112 | 0.079 | 0.103 |
| H_Lin_ | -0.010 | **-0.122** | -0.002 | **0.217** | **0.086** | -0.022 | **-0.131** | **0.142** | **0.077** | -0.042 | -0.007 | **0.082** | 0.050 |  | -0.041 | 0.060 | -0.127 | -0.024 |
| I_c_ | -0.032 | -0.056 | 0.034 | **-0.096** | 0.036 | -0.007 | -0.020 | 0.042 | -0.045 | 0.002 | **0.114** | 0.043 | **-0.073** | 0.032 |  | -0.129 | 0.016 | -0.115 |
| S_c_ | 0.015 | -0.030 | 0.060 | -0.064 | 0.039 | 0.032 | -0.045 | 0.043 | 0.024 | 0.041 | **0.069** | -0.054 | -0.002 | -0.039 | **-0.170** |  | 0.007 | 0.015 |
| τ | **0.126** | 0.032 | 0.029 | 0.053 | 0.057 | -0.021 | -0.037 | -0.010 | -0.003 | 0.051 | <0.001 | -0.020 | -0.017 | 0.032 | 0.004 | 0.041 |  | 0.131 |
| δ | 0.004 | **-0.129** | 0.002 | **-0.210** | -0.015 | -0.006 | -0.014 | 0.021 | 0.065 | -0.038 | 0.062 | 0.005 | 0.055 | -0.003 | -0.042 | -0.013 | -0.023 |  |

Cell formatting reflects significant correlations (bold text), correlation coefficient sign changes between the two scenarios (bordered cells), and more than two fold magnitude changes in the correlation coefficients between the two scenarios (blue cells indicate the correlation was stronger in scenario 1 and red cells indicate the correlation was stronger in scenario 3).

### Table 2. Spearman parameter correlations for scenarios 1 (lower left triangle) and 3 (upper right triangle) for Peneng, PNG.

|  | λ | α | k_0_ | k_Lin_ | κ | r | σ | Ψ_1_ | Ψ_2_ | μ | γ | g | c | H_Lin_ | I_c_ | S_c_ | τ | δ |
| --- | --- | --- | --- | --- | --- | --- | --- | --- | --- | --- | --- | --- | --- | --- | --- | --- | --- | --- |
| λ |  | **-0.062** | -0.016 | -0.025 | 0.018 | **-0.064** | **0.089** | **-0.093** | **-0.047** | **0.056** | 0.010 | -0.021 | 0.014 | -0.030 | -0.017 | -0.008 | -0.005 | -0.024 |
| α | **-0.064** |  | -0.033 | **-0.062** | 0.029 | **-0.182** | **0.095** | **-0.100** | **-0.186** | **0.150** | **0.123** | **-0.125** | 0.015 | <0.001 | **-0.102** | **-0.076** | -0.012 | **-0.080** |
| k_0_ | -0.013 | -0.023 |  | 0.011 | 0.002 | 0.037 | 0.017 | -0.029 | **0.044** | 0.002 | -0.024 | 0.007 | 0.023 | -0.033 | 0.036 | -0.013 | **0.044** | 0.009 |
| k_Lin_ | -0.018 | **-0.049** | 0.001 |  | **-0.090** | **0.139** | 0.023 | -0.002 | **-0.045** | 0.028 | **0.046** | -0.024 | **0.057** | 0.023 | **-0.206** | **-0.062** | 0.033 | **-0.113** |
| κ | **0.079** | **0.088** | -0.011 | 0.004 |  | **0.062** | 0.007 | 0.017 | 0.010 | -0.028 | -0.011 | 0.012 | <0.001 | <0.001 | -0.010 | -0.020 | 0.002 | -0.032 |
| r | **-0.098** | **-0.099** | -0.023 | 0.006 | **0.154** |  | **0.065** | **-0.086** | **-0.180** | **0.105** | 0.019 | **-0.066** | **0.047** | 0.034 | **-0.069** | -0.016 | 0.042 | **-0.058** |
| σ | **-0.055** | **-0.181** | 0.012 | **-0.043** | **0.096** | **-0.079** |  | **0.133** | **0.127** | **-0.061** | -0.040 | **0.053** | **-0.060** | -0.012 | **0.059** | 0.009 | -0.028 | 0.041 |
| Ψ_1_ | **0.055** | **0.142** | <0.001 | **0.030** | **-0.077** | **0.054** | **0.113** |  | **-0.077** | 0.035 | **0.046** | **-0.042** | **0.046** | 0.019 | **-0.056** | 0.001 | **0.042** | **-0.043** |
| Ψ_2_ | 0.022 | **0.113** | -0.022 | **0.044** | **-0.053** | **0.041** | **0.100** | **-0.056** |  | **0.091** | **0.111** | **-0.094** | 0.038 | **0.046** | -0.015 | **-0.067** | 0.022 | **-0.101** |
| μ | **-0.031** | **-0.133** | 0.005 | **-0.033** | **0.040** | **-0.057** | **-0.095** | **0.052** | **0.037** |  | **-0.076** | **0.059** | -0.025 | **-0.051** | 0.026 | 0.025 | **-0.043** | **0.081** |
| γ | 0.015 | **0.043** | -0.001 | **0.069** | **-0.052** | 0.028 | **0.046** | **-0.036** | **-0.036** | 0.016 |  | **0.043** | **-0.043** | -0.020 | **0.045** | 0.025 | 0.035 | **0.064** |
| g | 0.003 | **0.036** | -0.014 | 0.020 | -0.019 | 0.004 | 0.030 | -0.023 | -0.027 | 0.026 | -0.022 |  | **0.043** | **0.053** | -0.009 | -0.013 | **0.052** | **-0.061** |
| c | 0.020 | 0.018 | -0.001 | **-0.096** | 0.008 | 0.001 | 0.007 | -0.011 | -0.015 | **0.036** | <0.001 | -0.017 |  | -0.013 | 0.021 | 0.017 | -0.003 | -0.024 |
| H_Lin_ | **-0.057** | **-0.167** | 0.024 | **0.130** | **0.071** | **-0.088** | **-0.171** | **0.106** | **0.037** | **-0.076** | **0.043** | **0.040** | **0.055** |  | -0.011 | 0.004 | -0.015 | -0.008 |
| I_c_ | -0.006 | **-0.118** | 0.027 | **-0.213** | **0.061** | **-0.045** | -0.027 | **0.033** | **0.036** | -0.008 | 0.029 | 0.012 | -0.005 | **-0.048** |  | -0.039 | 0.010 | -0.015 |
| S_c_ | 0.002 | **-0.056** | -0.003 | **-0.100** | -0.001 | 0.003 | **-0.068** | 0.014 | 0.029 | -0.025 | 0.002 | 0.001 | -0.010 | **-0.042** | **-0.035** |  | 0.034 | -0.020 |
| τ | -0.003 | -0.006 | 0.022 | 0.021 | -0.010 | 0.027 | **0.037** | -0.023 | <0.001 | 0.027 | -0.014 | -0.022 | -0.003 | **0.033** | 0.019 | **0.047** |  | 0.009 |
| δ | **-0.030** | **-0.083** | -0.001 | **-0.109** | **0.049** | **-0.045** | **-0.100** | **0.067** | **0.064** | **-0.033** | -0.015 | 0.015 | -0.026 | **-0.060** | -0.021 | -0.019 | 0.009 |  |

Cell formatting reflects significant correlations (bold text), correlation coefficient sign changes between the two scenarios (bordered cells), and more than two fold magnitude changes in the correlation coefficients between the two scenarios (blue cells indicate the correlation was stronger in scenario 1 and red cells indicate the correlation was stronger in scenario 3).

As provided in Table 3 in the main manuscript for Alagramam, the pairwise parameter correlations are given for scenario 1 (baseline only) and scenario 3 (baseline + post-MDA 3 + post-MDA 5) for Kirare and Peneng in the Tables above. For Kirare, there are 39 significant correlations in scenario 1 versus 16 in scenario 3. The strongest correlations are seen with parameters relating to immunity (c, δ, $I_{C}$), larval establishment ($\psi_{2}$), infection aggregation ($k_{Lin}$), mf production (α), and mf and adult worm mortality (γ,μ). As for Alagramam, the sign and magnitude of correlations between these key model parameters are found to change between the two tested data scenarios. For Peneng, there are 78 significant correlations in scenario 1 versus 68 in scenario 3. The strongest correlations are seen with parameters relating to immunity ($I_{C}$), larval establishment ($\psi_{2}$), infection aggregation ($k_{Lin}$), mf production (α), and mosquito and adult worm mortality (σ,μ). Again, the sign and magnitude of these inter-parameter correlations changed between the two scenarios.
